# IFNγ Enhances CD64-Potentiated Phagocytosis of *Treponema pallidum* Opsonized with Human Syphilitic Serum by Human Macrophages

**DOI:** 10.3389/fimmu.2017.01227

**Published:** 2017-10-05

**Authors:** Kelly L. Hawley, Adriana R. Cruz, Sarah J. Benjamin, Carson J. La Vake, Jorge L. Cervantes, Morgan LeDoyt, Lady G. Ramirez, Daniza Mandich, Mary Fiel-Gan, Melissa J. Caimano, Justin D. Radolf, Juan C. Salazar

**Affiliations:** ^1^Department of Pediatrics, UConn Health, Farmington, CT, United States; ^2^Division of Infectious Diseases, Connecticut Children’s Medical Center, Hartford, CT, United States; ^3^Centro Internacional de Entrenamiento e Investigaciones Médicas (CIDEIM), Cali, Colombia; ^4^Department of Immunology, UConn Health, Farmington, CT, United States; ^5^Department of Medicine, UConn Health, Farmington, CT, United States; ^6^Department of Pathology, Hartford Hospital, Hartford, CT, United States; ^7^Department of Genetics and Developmental Biology, UConn Health, Farmington, CT, United States

**Keywords:** *Treponema pallidum*, human, macrophage, Fcγ receptor, phagocytosis, phagosomal signaling, vaccine model

## Abstract

Syphilis is a multi-stage, sexually transmitted disease caused by the spirochete *Treponema pallidum* (*Tp*). Considered broadly, syphilis can be conceptualized as a dualistic process in which spirochete-driven inflammation, the cause of clinical manifestations, coexists to varying extents with bacterial persistence. Inflammation is elicited in the tissues, along with the persistence of spirochetes to keep driving a robust immune response while evading host defenses; this duality is best exemplified during the florid, disseminated stage called secondary syphilis (SS). SS lesions typically contain copious amounts of spirochetes along with a mixed cellular infiltrate consisting of CD4^+^ T cells, CD8^+^ T cells, NK cells, plasma cells, and macrophages. In the rabbit model, *Tp* are cleared by macrophages *via* antibody-mediated opsonophagocytosis. Previously, we demonstrated that human syphilitic serum (HSS) promotes efficient uptake of *Tp* by human monocytes and that opsonophagocytosis of *Tp* markedly enhances cytokine production. Herein, we used monocyte-derived macrophages to study *Tp*–macrophage interactions *ex vivo*. In the absence of HSS, monocyte-derived macrophages internalized low numbers of *Tp* and secreted little cytokine (e.g., TNF). By contrast, these same macrophages internalized large numbers of unopsonized *Borrelia burgdorferi* and secreted robust levels of cytokines. Maturation of macrophages with M-CSF and IFNγ resulted in a macrophage phenotype with increased expression of HLA-DR, CD14, inducible nitric oxide synthase, TLR2, TLR8, and the Fcγ receptors (FcγR) CD64 and CD16, even in the absence of LPS. Importantly, IFNγ-polarized macrophages resulted in a statistically significant increase in opsonophagocytosis of *Tp* accompanied by enhanced production of cytokines, macrophage activation markers (CD40, CD80), TLRs (TLR2, TLR7, TLR8), chemokines (CCL19, CXCL10, CXCL11), and T_H_1-promoting cytokines (IL-12, IL-15). Finally, the blockade of FcγRs, primarily CD64, significantly diminished spirochetal uptake and proinflammatory cytokine secretion by IFNγ-stimulated macrophages. Our *ex vivo* studies demonstrate the importance of CD64-potentiated uptake of opsonized *Tp* and suggest that IFNγ-activated macrophages have an important role in the context of early syphilis. Our study results also provide an *ex vivo* surrogate system for use in future syphilis vaccine studies.

## Introduction

Venereal syphilis is a multistage, sexually transmitted infection caused by the spirochetal bacterium, *Treponema pallidum* (*Tp*) (1, 2). Syphilis continues to be a major public health threat, affecting nearly 6 million people globally (3). In the United States, the rate of primary and secondary syphilis (SS) has more than tripled from 2.1 cases in 2000 to 6.3 cases per 100,000 in 2014 (4, 5). Syphilitic infection commences clinically with the appearance of an ulcerative lesion, known as a chancre, at the site of inoculation (2). However, even before the appearance of the chancre, treponemes begin to disseminate hematogenously, eventually giving rise to SS, a systemic inflammatory illness associated with diverse clinical manifestations, most commonly involving skin and mucous membranes (2). After weeks to months, the robust cellular and humoral responses elicited during SS gain control of the pathogen, driving down spirochetal burdens and ushering in the asymptomatic stage called latency (2). Approximately one-third of untreated patients will develop one of the recrudescent syndromes collectively known as tertiary syphilis (2). Considered broadly, syphilis can be conceptualized as a dualistic process in which spirochete-driven inflammation, the cause of clinical manifestations, coexists to varying extents with bacterial persistence.

Syphilitic lesions in all stages of disease contain a rich cellular infiltrate, composed primarily of lymphocytes, plasma cells and macrophages, accompanied by vasculopathic changes of varying severity (6, 7) capable of giving rise to a wide variety of histological patterns, including granulomata (8). Immunocytochemical analysis has revealed that the relative proportions of T cell subsets shifts from predominantly CD4^+^ T cells in genital ulcers to a predominance of CD8^+^ T cells in SS lesions (7, 9). Innate lymphocytes, more specifically NK cells, also have been identified in biopsies of skin rashes from SS patients (10). Transcriptional and immunofluorescent analyses have revealed that both primary and SS lesions contain classic T_H_1 cytokines and that multiple lymphocytic populations (CD4^+^, CD8^+^, and NK cells) can be sources of IFNγ, the hallmark of a T_H_1 response (10–14). The presence in human lesions of IFNγ and activated macrophages (9, 14) is consistent with a large body of literature from the experimental rabbit model suggesting that macrophages activated by IFNγ (15–18) are critical for spirochete clearance. Seminal *ex vivo* studies by Lukehart et al. (19–22) have demonstrated that opsonic antibodies present in immune rabbit serum markedly enhance phagocytosis and killing of *Tp* by rabbit peritoneal macrophages. Like their rabbit counterparts, human monocytes and macrophages also require syphilitic serum for efficient spirochetal uptake (10, 23). In addition to clearance, opsonophagocytosis of *Tp* also promotes a robust proinflammatory cytokine response which can be attributed to the release of spirochetal pattern-associated molecular patterns (PAMPs), most notably lipoproteins, from organisms degraded in phagosomes (10).

Despite the acknowledged central role of the macrophage in the immunobiology of syphilis, only recently have investigators begun to examine the interactions between *Tp* and human macrophages. We, like others, observed *Tp* infiltrating the epidermis by immunohistologic analysis of secondary syphilitic skin lesions (24, 25). Additionally, CD68^+^ histiocytes were poised throughout the dermis, at the dermal-epidermal juncture and, in some cases, a few histiocytes were observed in the epidermis. CD138^+^ plasma cells, a potential source of local opsonic antibodies, made up a portion of the cell-mediated inflammatory response (26). As in the recent communication by Marra et al. (23), herein we report that opsonic antibodies significantly enhance internalization of *Tp* by monocyte-derived human macrophages. However, we also note that IFNγ markedly enhances the expression of CD64, the primary receptor for opsonic uptake of treponemes, providing for the first time, mechanistic evidence for the linkage of spirochetal clearance with adaptive cellular responses *in vivo*. IFNγ plays a critical role in macrophage mediated responses by not only increasing opsonophagocytosis of *Tp* but also markedly broadening the inflammatory response of the macrophages following internalization of spirochetes, further emphasizing the interdependence of local innate and adaptive responses during syphilitic infection. However, even with IFNγ activation, uptake of *Tp* by macrophages was inefficient as determined by the substantial proportion of spirochetes that were not internalized despite a lengthy preincubation in high concentrations of the anti-treponemal antibodies present in human syphilitic sera (HSS). These latter results are in accord with prior studies showing that *Tp* presents a paucity of surface antigenic targets at the host-pathogen interface and that spirochete populations display a high degree of heterogeneity with respect to surface antibody (Ab) binding. Our findings not only illustrate the dualistic nature of syphilis but also demonstrate the utility of an *ex vivo* model for teasing apart its many components.

## Materials and Methods

### Ethics Statement

This study was carried out in accordance with the recommendations of the Institutional Review Boards at UConn Health, Farmington CT and Centro Internacional de Entrenamiento e Investigaciones Médicas (CIDEIM) in Cali, Colombia. All study participants were provided written informed consent. All animal experimentation was conducted following the *Guide for the Care and Use of Laboratory Animals* (8th Edition) and in accordance with protocols reviewed and approved by the UConn Health Institutional Animal Care and Use Committee under the auspices of Animal Welfare Assurance A347-01.

### Immunohistochemical (IHC) Analysis of SS Skin Biopsies

Four micrometer sections, cut from a 4-mm punch biopsy that were fixed in 10% buffered formalin and embedded in paraffin, were stained with hematoxylin and eosin (H&E) or labeled immunohistochemically with antibodies against CD4 (clone EP204, Epitomics, Cambridge, MA), CD8 (clone 4B11, Leica Biosystems Inc., Buffalo Grove, IL), CD56 (clone 56C04 Thermo-Scientific, Waltham, MA), CD68 (clone PG-M1, Dako, Carpinteria, CA) and CD138 (clone B-A38 Cell Marque, Rocklin, CA) using an automated immunohistochemistry staining platform (Bond Max, Leica-Microsystems, Buffalo Grove, IL). IHC detection of *Tp* was performed manually as previously described (10) using a rabbit polyclonal anti-*Tp* Ab (Biocare, Concord, CA, USA). Skin specimens from healthy volunteers of the same socioeconomic background and conditions were not available for analysis. Tissue known to contain *Tp* were used for the positive control of *Tp* IHCs. Additionally, secondary alone controls were used to assess any non-specific Ab binding for each Ab used.

### Bacterial Strains

*Treponema pallidum* (Nichols strain) was propagated by intratesticular inoculation of adult male New Zealand white rabbits and harvested in CMRL medium (Gibco) supplemented with 10% fetal bovine serum (FBS) (Atlantic Biologicals, Miami, FL, USA) at peak orchitis (12). Spirochetes were enumerated by dark-field microscopy on a Petroff-Hausser counting chamber (Hausser Scientific, Horsham, PA, USA). Virulent *Borrelia burgdorferi* (*Bb*) strain 297 encoding green fluorescent protein on a cp32-based shuttle vector (27) was propagated in BSK-H medium containing 6% rabbit serum (Sigma-Aldrich Chemical Co., St. Louis, MO, USA) and 400 μg/ml of kanamycin (Sigma-Aldrich Chemical Co.). For macrophage incubation experiments, *Bb* temperature-shifted from 23 to 37°C were grown to late-logarithmic phase (~8 × 10^7^ spirochetes/ml), washed twice with RPMI, and resuspended in RPMI to a final density of ~3 × 10^8^ spirochetes/ml.

### Macrophage Maturation and Activation

Peripheral blood was obtained from healthy donors determined to be seronegative for syphilis by Rapid Plasma Reagin test and/or Lyme disease by enzyme-linked immunosorbent assay (ELISA) performed in the clinical laboratory at John Dempsey Hospital. Peripheral blood mononuclear cells (PBMCs) were isolated using Lymphoprep and SepMate-50 tubes in accordance with the recommendations of the manufacturer (STEMCELL Technologies, Vancouver, BC, Canada). To generate all macrophage phenotypes, 3 × 10^7^ PBMCs were plated in 10 cm polystyrene Petri dishes (BD Falcon) and incubated for 2 h at 37°C with 5% CO_2_. Adherent cells were washed thoroughly with ice-cold PBS to remove non-monocyte populations. Macrophage nomenclature used throughout follows the recommendations of Murray et al. (28). MΦ(−)s were generated by incubating the adherent monocytes in RPMI-1640 (RPMI) medium (Gibco, Thermo-Scientific) supplemented with 20% heat-inactivated (56°C for 30 min) normal human serum (NHS) (CORNING, Corning, NY, USA) and 1% penicillin–streptomycin (10,000 U/ml) (Gibco) for 10 days. MΦ(C)s were generated by incubating the adherent monocytes in RPMI, 20% heat-inactivated NHS, and 50 ng/ml of M-CSF (Peprotech, Rocky Hill, NJ, USA) (29) for 10 days. To generate MΦ(IFNγ)s, MΦ(C)s were divided on day 7 and 2.5 ng/ml of recombinant IFNγ (Roche Diagnostics, Mannheim, Germany) (30) was added to one portion for an additional three days of incubation. Media and cytokines were replenished every 3 days for a total of 10 days. Because *Tp* lacks endotoxin and would not be encountered by macrophages in syphilitic lesions, LPS, which is often used in macrophage differentiation protocols, was omitted from all culture media.

### Human Syphilitic Sera

All *Tp* opsonization studies were performed using a pool of deidentified sera collected from five HIV-negative primary or SS patients seen at Parkland Memorial Hospital in Dallas, TX, USA (approved for use by the Institutional Review Board of the UConn Health) (31). Strong reactivity of the pool with *Tp* proteins was confirmed by immunoblot (Trinity Biotech, Carlsbad, CA, USA, Figure S2 in Supplementary Material).

### *Tp* Opsonophagocytosis Assays

Freshly harvested *Tp*, adjusted to a final concentration of 3 × 10^8^ treponemes/ml, were incubated with either 10% NHS or 10% pooled HSS for 2 h at RT prior to addition to macrophages. Macrophages, differentiated as described above, were plated at 1 × 10^5^ cells in 500 μl of RPMI supplemented with 10% heat-inactivated FBS (Hyclone Laboratories, Inc., Logan, UT, USA) in an eight-well chamber microscopy slide (Millipore, Billerica, MA, USA). *Tp* were added at a multiplicity of infection (MOI) of 30:1 for 8 h at 37°C with 5% CO_2_. All culture media and reagents were confirmed to be free of LPS contamination (≤10 pg/ml) by Limulus amebocyte lysate assay quantification (Cambrex, MA, USA). Following incubation, supernatants were removed and macrophages were prepared for IFA to evaluate binding and uptake of treponemes. Cells were fixed and permeabilized with 2% paraformaldehyde and 0.01% Triton X-100 for 10 min at RT. They then were rinsed with PBS, blocked with PBS containing 10% normal goat serum (NGS) for 1 h at RT, and then incubated with rabbit polyclonal anti-*Tp* (1:100, Abcam, Cambridge, MA, USA) in PBS 1% NGS for 1 h at RT. After four successive washes with PBS, the cells were then incubated with goat anti-rabbit immunoglobulin G (IgG)-Texas Red (1:500) in PBS 1% NGS for 1 h at RT. After staining for *Tp*, actin cytoskeletons were stained with Phalloidin-AF488 (1:20) (Life Technologies, Carlsbad, CA, USA) for 20 min at RT. The cells were then washed thoroughly with PBS six times, rinsed with deionized water to remove salt and allowed to air dry. Finally, Vectashield containing DAPI (Vector Laboratories, Inc., Burlingame, CA, USA) was added and samples were sealed with a coverslip. To assess binding and internalization of *Tp*, images of 100–200 macrophages were acquired on an Olympus BX60 epifluorescence microscope equipped with a Retiga 2000R CCD camera (QImaging) or Ziess LSM 780 confocal microscope mounted on an inverted Axio Observer Z1. Similar numbers of macrophages were imaged for each experimental condition per biological replicate. Acquired images were processed with ImageJ (version 1.5.1 g) (NIH, USA) and uptake was quantitated in a blinded fashion. The percentage of macrophages with bound spirochetes was quantified by dividing the number of cells with surface bound spirochetes by the total number of macrophages imaged for each condition. The percentage of spirochete-positive macrophages were calculated by dividing the number of cells containing ≥1 internalized spirochetes by the total number of cells imaged. Phagocytic indices were calculated by dividing the number of degraded, internalized spirochetes by the total number of spirochete-positive macrophages for the same condition. To quantify the percentage of *Tp* remaining after the 8 h incubation period, 10 μl aliquots from *Tp*-stimulated-macrophage supernatants were enumerated, in triplicate, by dark-field microscopy. Percentage of *Tp* recovered was calculated using a “time zero” spirochetal count.

### *Bb* Phagocytosis Assays

MΦ(−)s, after maturation as described above, were plated at 1 × 10^5^ cells in 500 μl of RPMI supplemented with 10% FBS in an eight-well chamber microscopy slide and incubated at 37°C with 5% CO_2_ overnight to allow for attachment. Prior to incubation, medium was removed and replaced with fresh RPMI supplemented with 10% FBS. *Bb* were added at an MOI of 30:1 and incubated for 8 h. Following incubation, cells were fixed and permeablized and cytoskeletons were stained with Phalloidin-AF594 as described above. Binding and internalization of spirochetes was performed as described above.

### Cytokine Analysis

TNF, IL-6, IL-1β, IL-10, IL-12, and IL-8 were measured in supernatants using a Human Inflammatory Cytokine Bead Array per the manufacturer’s (BD Biosciences, San Jose, CA, USA) protocol. Data were collected on a MACSQaunt Analyzer (Miltenyi Biotec, Germany) and analyzed with FCAP Array™ Software version 3.0 (BD Biosciences).

### Flow Cytometry

MΦ(C)s and MΦ(IFNγ)s were harvested from 10-cm polystyrene Petri dishes by replacing growth medium with PBS containing 1% FBS and gently lifting the cells with a scraper. The macrophages were washed once with PBS in 50 ml conical tubes by spinning at 300*g* for 10 min; the cells were decanted, resuspended in PBS 1% FBS, and dispensed into FACS tubes in preparation for staining. Cells were incubated for 10 min at 4°C with 10 μg/ml of purified human IgG (Sigma, St. Louis, MO, USA) for Fcγ receptor (FcR) blocking, followed by a 20 min incubation with fluorochrome-conjugated antibodies. Antibodies obtained from Biolegend (CD32-APC, CD64-PECy7, CD163-PerCP/Cy5.5, CD206-APC, anti-rabbit IgG-BV421), eBioscience (CD14-APC, CD16-PE, TLR2-FITC, TLR4-APC), Novus Biologicals (TLR7-PE, TLR8-AF647), BD Biosciences (HLA-DR-PE), Invitrogen (CD68-FITC), and Abcam [rabbit anti-human inducible nitric oxide synthase (iNOS)] were used at dilutions recommended by the manufacturers. For intracellular staining, the cells were surface-stained as described above, permeabilized in 250 μl of Cytofix/Cytoperm solution (BD Biosciences) for 20 min at 4°C, washed with PermWash buffer (BD Biosciences), stained with fluorochrome-conjugated antibodies (diluted in 50 μl of PermWash buffer) for 30 min at 4°C, and washed twice with PermWash and resuspended in FACS buffer. A minimum of 10,000 single cells events were acquired using a BD LSR-II flow cytometer and FACSDIVA™ software (BD Biosciences). Analysis of immunostaining was performed using FlowJo V10 for Mac (FlowJo LLC, Ashland, OR, USA). Mean fluorescence intensity (MFI) values were determined after subtracting background fluorescence (32).

### Targeted Array Analysis

1 × 10^6^ MΦ(C)s and MΦ(IFNγ)s were plated in 1 ml of RPMI containing 10% FBS in a 12-well tissue culture plate (Corning) and incubated with unopsonized or opsonized *Tp* for 8 h at an MOI 30:1. RNAs were extracted using a NucleoSpin RNA purification kit according to the manufacturer’s (Macherey-Nagel Inc., Bethlehem, PA, USA) instructions and their concentrations determined using a Nanodrop spectrophotometer (Thermo-Scientific). cDNA synthesis was performed using the High Capacity cDNA Archive Kit (Applied Biosystems, Waltham, MA, USA) according to the manufacturer’s instructions. Transcripts were amplified using TaqMan^®^ Human Immune Array (384-fluidics card) and TaqMan^®^ Human Phagocytosis Array (96-well plate) per manufacturer’s instructions (Applied Biosystems). Briefly, the Immune Array was performed in a 2 μ1 reaction volume containing 62.5 pg of cDNA, and 1 μ1 of gene expression master mix. The Phagocytosis Array was processed in a 20 μl reaction volume containing 5 ng of cDNA and 10 ml of gene expression master mix. Commercially available primers (Applied Biosystems) were used to amply transcripts for human *IRF3* (Hs01547283_m1), *IRF5* (Hs00158114_m1), *IRF7* (Hs00185375_m1), and *IFNB* (Hs00277188_s1) as described previously (33). Amplification reactions were performed using a 7900HT Fast Real Time thermocycler (Applied Biosystems) using the following conditions: 95°C for 20 min, and 40 cycles of 95°C for 1 s and 60°C for 20 s. Expression levels for all transcripts were normalized to *GAPDH* and the relative changes in gene expression between experimental groups were calculated using the 2^−ΔΔCt^ method (34). To identify genes differentially expressed in the presence of IFNγ and absence of *Tp*, the average fold changes for unstimulated MΦ(IFNγ)s were calculated relative to the unstimulated MΦ(C)s. To identify genes differentially expressed in the presence of *Tp*, relative fold changes were calculated based on the unstimulated MΦ(IFNγ)s condition, with a two-fold change and *p*-value < 0.05 threshold (Student’s *t*-test) (35). Heat maps and graphs of the fold changes of transcript expression were generated using R statistical software, version 3.2.2 with package “ggplot2,” function “heatmap.2.” Fold change values displayed in the heat maps represent the average fold change compared to the corresponding unstimulated MΦ(IFNγ). No clustering analysis was performed.

### Colocalization of CD64 and *Tp*

1 × 10^5^ MΦ(IFNγ)s were plated in 500 μl of RPMI supplemented with 10% FBS in an eight-well chamber microscopy slide and incubated for 8 h with *Tp* at an MOI of 30:1. Following incubation, the cells, fixed and permeablized as above, were incubated with mouse anti-human CD64 (1:25, clone 10.1, Abcam) and rabbit polyclonal anti-*Tp* 1 h at RT, and then washed four times with PBS followed by incubation for 1 h at RT with goat anti-mouse IgG-OregonGreen488 (1:200, Invitrogen, Carlsbad, CA, USA) and goat anti-rabbit IgG-Texas Red (1:500, Life Technologies). Secondary Ab alone controls were evaluated for non-specific binding (Figure S6 in Supplementary Material). Images were acquired on a Zeiss LSM 780 confocal microscope mounted on an inverted Axio Observer Z1 and processed with ImageJ as described above. Colocalization of *Tp* and CD64 was visualized using the ImageJ plugin “Colocalization”; the coefficient of colocalization was determined using the plugin “JACoP.”

### FcγR Blocking Experiments

MΦ(IFNγ)s were pre-incubated with 10 μg/ml of mouse antihuman CD64 (clone 10.1, Abcam), mouse anti-human CD32 (clone 6C4, Affymetrix eBiosciences, San Diego, CA, USA), mouse anti-human CD16 (clone 3G8, Biolegend, San Diego, CA, USA), or 10 μg/ml each of all three antibodies for 1 h prior to adding spirochetes. Opsonophagocytosis assays were performed as described above.

### Statistics

General statistical analysis was conducted using GraphPad Prism 6.0 h (GraphPad Software, San Diego, CA, USA). Phagocytic uptake/index, *Tp* recovery counts, cytokine concentrations, cytometric MFI ratio, and fold increase or decrease for each gene transcript assayed were compared among the different stimuli. Either a paired or unpaired Student’s *t*-test (i.e., Mann–Whitney test or Wilcoxon test) was used for comparison across two groups. For the analysis of three or more conditions, we used a non-parametric statistical test for trend analysis (Friedman’s test with a Dunnett’s multiple comparisons post-test analysis). For each experiment, the standard error of the mean was calculated and a *p*-value < 0.05 was considered significant.

## Results

### *T. pallidum*-Macrophage Interactions in SS Skin Lesions

**Figure 1:**
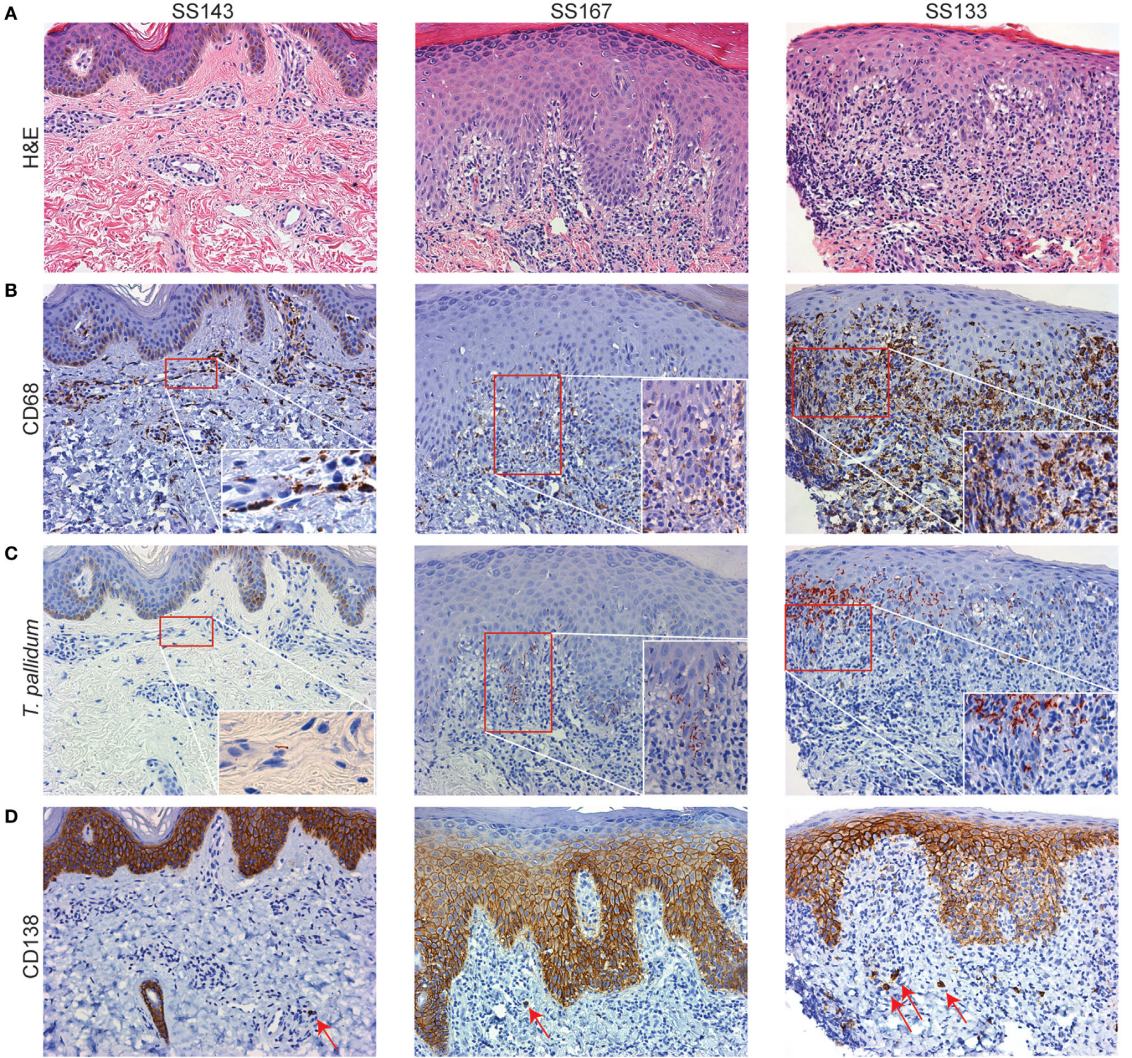
Secondary syphilis (SS) skin lesions are enriched with dermal macrophages. Representative skin biopsies obtained from three SS patients’ skin lesions were processed for hematoxylin and eosin (H&E) and immunohistochemical (IHC) analysis. **(A)** H&E-stained sections (10× magnification) reveal an extensive dermal–epidermal lymphohistiocytic immune cell infiltrate with perivascular inflammatory changes. SS167 reveals mild psoriasiform hyperplasia with basal vacuolar changes. SS133 exhibits a classic lichenoid reaction pattern with basal vacuolation. **(B–D)** IHC testing of sequential tissue sections **(B)** varying numbers of CD68^+^ macrophages were observed in various locations throughout the dermis, but very few were present in the epidermis; **(C)** *Treponema pallidum* is shown in clusters and across the dermal–epidermal barrier (20× magnification). Insets depict higher magnifications of the red box areas in **(B,C)**. **(D)** CD138^+^ plasma cells (red arrows) varied in frequency and location, including the perivascular space and throughout the papillary dermis. CD138 in basal epidermis has been documented but is non-contributory.

To set the stage for our *ex vivo* studies, we assessed the distribution of macrophages, lymphocytes, and plasma cells in spatial relationship to *Tp* within representative skin punch biopsy specimens obtained from three HIV-negative SS patients. In line with prior studies (6, 7, 10, 36–39), IHC analysis of SS skin biopsies revealed a rich dermal-epidermal infiltrate (Figure 1A), which in addition to CD4^+^, CD8^+^, and CD56^+^ lymphocytes (Figure S1 in Supplementary Material), had large numbers of CD68^+^ macrophages (40) (Figure 1B). Abundant CD68^+^ cells were present throughout the dermis, at the dermal–epidermal juncture, and, in some cases, surrounding vascular structures within the dermis (Figure 1B). Treponemal numbers and locations in the inflamed skin, as well as their spatial proximity with dermal macrophages, varied among samples (Figure 1C). In SS143 skin biopsy, a small cluster of CD68^+^ macrophages was visualized in close proximity to a spirochete surrounding a blood vessel wall. In SS167 sample, substantial numbers of spirochetes were seen within the interstitium of the papillary dermis (Figures 1B,C). In SS133, large groups of spirochetes also were observed traversing the dermal–epidermal junction and within the stratum basale and stratum spinosum of the epidermis (Figure 1C), whereas very few macrophages were found within the epidermis (Figure 1B). Of particular interest, CD138^+^ plasma cells were commonly observed in perivascular locations and throughout the papillary dermis (Figure 1D). In concert with previously published studies (2, 6, 7), our current results confirm the presence of macrophages in *Tp* infected tissue, and suggest that the cellular environment may be involved in shaping the phenotype of macrophages, in addition to providing a local source of opsonic antibodies needed for spirochetal recognition and clearance in *Tp* infected tissues.

### Primary Human Macrophages Phagocytose *Tp* in the Presence of HSS but Exhibit a Blunted Cytokine Output

**Figure 2:**
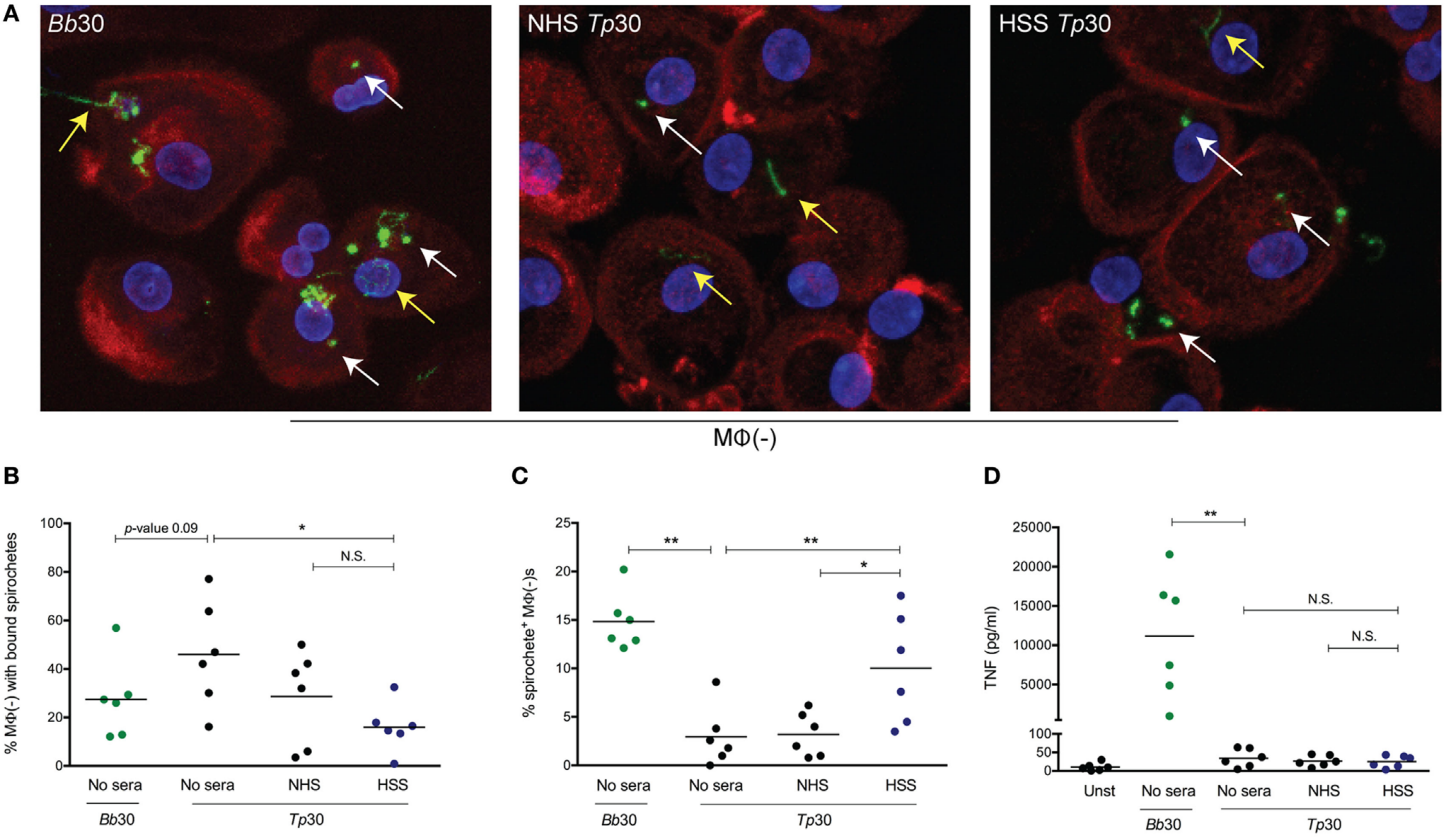
Spirochetal uptake and cytokine secretion by primary human macrophages. Monocyte-derived macrophages [depicted as MΦ(−)], matured with 20% normal human serum (NHS) were stimulated with *Borrelia burdorferi* (*Bb)* or *Treponema pallidum* (*Tp)* at an multiplicity of infection (MOI) 30:1 for 8 h. *Tp* were incubated alone (no sera) or, where indicated, with 10% heat inactivated NHS or human syphilitic serum (HSS). **(A)** Macrophage actin cytoskeleton (red), nucleus (blue), and *Bb* or *Tp* (green) were labeled and imaged as described in Section “Materials and Methods.” Representative confocal micrographs demonstrating binding (yellow arrow) and internalization (white arrow) of spirochetes are shown as 20 consecutive compressed Z-stack panels. Dot plot in **(B)** shows percentage of MΦ(−)s with surface bound spirochetes in four conditions, *Bb* alone and *Tp* with no sera, NHS, or HSS. Dot plot in **(C)** reveals the percentage of MΦ(−)s containing internalized *Bb* and *Tp* in the presence of HSS (blue circles) when compared to no sera and NHS (black circles). Dot plot in **(D)** reveals supernatant TNF concentration (pg/ml) for MΦ(−)s stimulated with *Bb* (green circles) and *Tp* under three different conditions. Statistical significance between *Bb* and no Sera *Tp* was determined by two-tailed Mann–Whitney test. The statistical significance between the three *Tp* conditions was determined by Friedman’s test with a Dunnett’s multiple comparison post-test analysis. N.S., not significant, *p-value of <0.05, **p-value of <0.01.

Having confirmed that the macrophage is an important cellular element of the inflammatory response to the syphilis spirochete *in vivo*, we next performed *ex vivo* experiments to directly characterize macrophage-*Tp* interactions. We began by maturing adherent human monocytes into macrophages using 20% heat-inactivated NHS without additional exogenous growth factors (e.g., M-CSF). We then assessed the ability of these macrophages [MΦ(−)] to bind and phagocytose spirochetes. As a phagocytosis control, we incubated MΦ(−)s with *Bb*, a spirochetal pathogen that is readily bound and internalized by primary human phagocytes (41, 42). As shown in Figure 2A and quantified in Figure 2B, *Bb* binds to MΦ(−)s and is internalized into phagosomal vacuoles (Figures 2A,C). We then assessed if macrophages were able to similarly bind and internalize *Tp* in the presence or absence of NHS or with HSS. For these experiments, we first confirmed that our pooled HSS reacted with spirochetal antigens by using a *Tp* IgG Marblot strip test (Figure S2 in Supplementary Material). The pooled HSS was determined to be highly reactive with many of *Tp*’s proteins, likely including some opsonic targets. It is important to note that we previously demonstrated that the same pooled HSS recognizes the Nichols strain variant of L4, an immunodominant extracellular loop in the β-barrel of BamA and opsonic target (31).

As shown in Figure 2, *Tp* binds to macrophages with or without NHS. The observed binding does not cause a concomitant increase in bacterial uptake, which suggests that *Tp* attaches to a non-phagocytic receptor yet to be defined on the macrophage’s surface. On the other hand, addition of HSS caused a significant increase in spirochetal uptake (Figures 2A,C). In line with the increased uptake of opsonized *Tp*, fewer spirochetes were visualized bound to the macrophage surface (Figure 2B). Interestingly, while internalization of *Bb* resulted in copious secretion of TNF, opsonophagocytosis of *Tp* did not (Figure 2D). These findings confirm that HSS promotes opsonophagocytosis of *Tp* by human macrophages, but also raise the possibility that the milieu in which macrophages encounter spirochetes is an important determinant of macrophage activation and cytokine production following internalization of bacteria.

### IFNγ Induces a Classically Activated Immunophenotype in Human Macrophages

**Figure 3:**
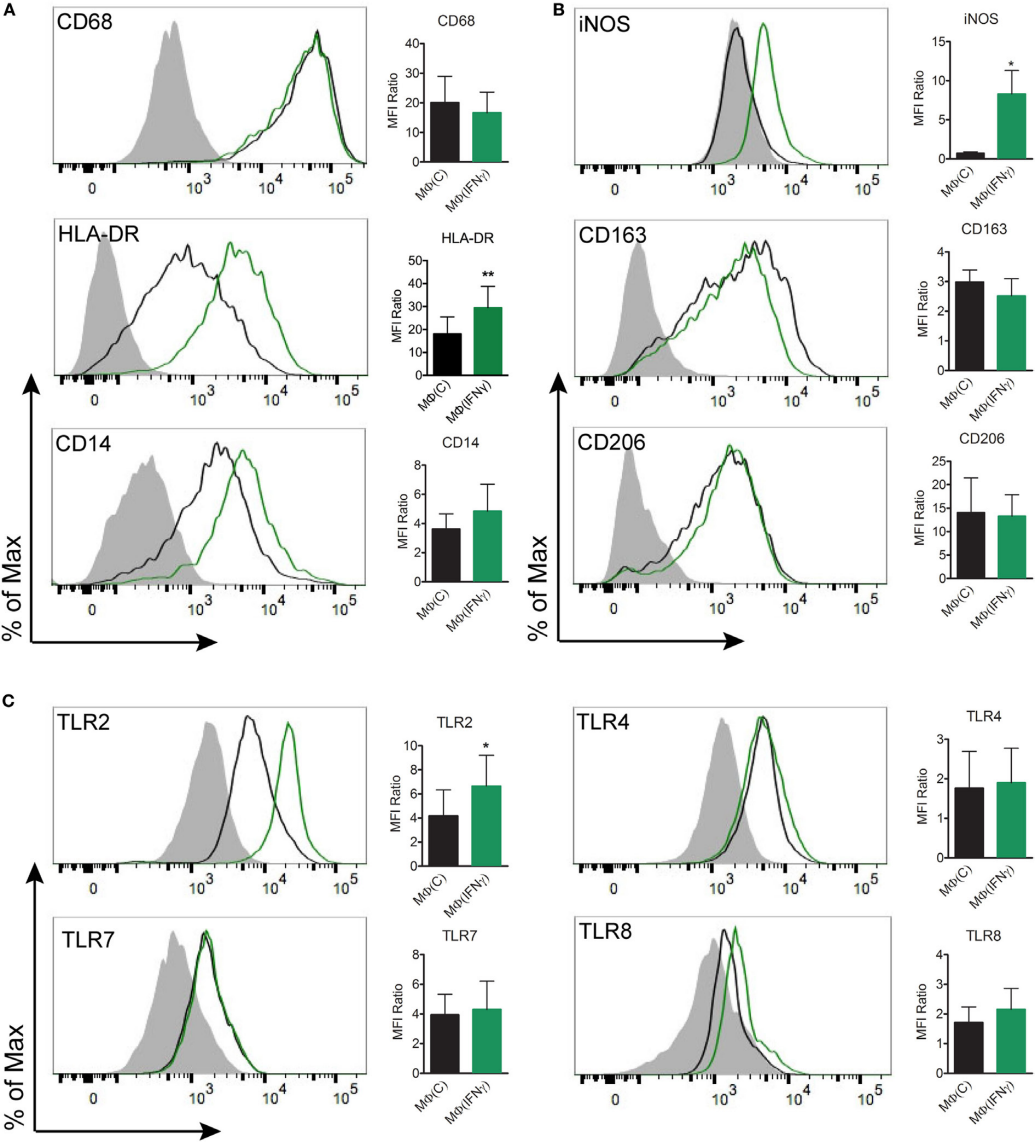
Flow cytometric analysis of macrophage immunophenotypic profile. Monocyte-derived macrophages were generated from healthy controls by incubating monocytes with M-CSF [MΦ(C)] for 7 days and then a portion of the macrophages were activated with IFNγ [MΦ(IFNγ)] for the remaining 3 days. MΦ(C)s (black) and MΦ(IFNγ)s (green) from the same participant were stained for **(A)** macrophage markers: CD68_IC_, HLA-DR, CD14, **(B)** phenotypic markers: iNOS_IC_, CD163, CD206, and **(C)** Toll-like receptors: TLR2, TLR4, TLR7_IC_, TLR8_IC_; the filled gray histograms indicate the isotype control. Histograms are representative of a minimum of four independent experiments that were used to quantify the mean fluorescence index (MFI) ratio ± the SD as described under Section “Materials and Methods.” Paired Student’s *t*-tests (Wilcoxon test) was used to determine statistical significance between the two macrophage phenotypes, *p-value of <0.05, **p-value of <0.01. IC, intracellular staining.

The observation that cytokine production in *Tp* stimulated MΦ(−)s was unexpectedly low prompted us to generate a macrophage phenotype that more closely resembles macrophages present within an IFNγ rich syphilitic immune microenvironment *in vivo* (10, 14). To do so, human monocytes were differentiated into macrophages by adding M-CSF alone [MΦ(C)] or M-CSF with IFNγ [MΦ(IFNγ)] and then characterized by flow cytometry, gene transcript analysis, and cytokine responsiveness. Given that *Tp* is completely devoid of LPS (43), we omitted this potent endotoxin from our maturation protocols, whereas LPS is often used in macrophage stimulation experiments (29, 44, 45). To more clearly define the effect of IFNγ, we first had to measure the expression of several markers associated with either classically and/or alternatively activated macrophages (29, 46, 47). Expression of CD68, a well characterized macrophage marker, was unaffected by IFNγ (Figure 3A), while expression of the antigen presentation molecule HLA-DR was appreciably increased (48) (Figure 3A). CD14, a glycosylphosphatidylinositol-linked membrane glycoprotein that interacts with several pattern recognition receptors (PRRs), including TLR2 and complement receptor-3 (CR3) (42, 49–51), was upregulated by IFNγ (Figure 3A). iNOS, notorious for its vital role in antimicrobial activity as part of the oxidative burst of macrophages, was also significantly increased by IFNγ (Figure 3B). We also assessed macrophage expression of the scavenger receptor CD163 (52) and the C-type lectin CD206 (29), which are associated with alternative macrophage activation. CD163 and CD206 were for the most part unaffected by IFNγ (Figure 3B). We then examined the expression of TLR2, 7, and 8, which we have shown to be upregulated in SS skin lesions (10). Expression of TLR7 was similar between the two macrophage phenotypes. On the other hand, TLR2, which is essential for spirochetal lipoprotein recognition, and TLR8, an endosomal TLR involved in single-stranded spirochetal RNA recognition (53), were increased in response to IFNγ (Figure 3C).

We further examined transcriptional differences between unstimulated MΦ(C)s and MΦ(IFNγ)s by using a commercially available microarray panel. As shown in Table 1, several genes that code for macrophage activation markers, microbial PRRs, cytokines and chemokines, were significantly upregulated by IFNγ. Among them, the activation markers *CD40* and *CD80*, as well as *CD38*, a type II transmembrane glycoprotein involved in Ca^2+^ signaling during FcγR-mediated bacterial uptake (54) were transcriptionally increased. In line with the flow cytometry results shown in Figure 3, *TLR2* and *TLR8* were also upregulated in the arrays. *PTGS2*, the gene which codes for COX-2 and is associated with classically activated macrophages (44), and several inflammatory chemokines and cytokines (*CXCL11, IL1B, IL1A, IL8, IL15*) involved in cellular recruitment and activation were also transcriptionally upregulated. Of particular importance, all three FcγRs (CD16, CD32, and CD64) were upregulated in MΦ(IFNγ)s. In parallel stimulation experiments, we compared proinflammatory cytokine production in response to several TLR ligands (MMP, LPS, or R848) between MΦ(C)s and MΦ(IFNγ)s. We observed that, independently of the TLR ligand used, IFNγ primed macrophages produced significantly more cytokines than non-primed MΦ(C)s (Figure S3 in Supplementary Material). Our combined observations thus confirm that M-CSF cultivated macrophages in the presence of IFNγ exhibit a classically activated macrophage phenotype.

**Table 1:** Transcription profile of MΦ(IFNγ)s.

Gene transcript	Protein name	Average fold change
**Activation markers**		
*CD38*	CD38	97.7
*CD40*	CD40	2.0
*CD80*	CD80	3.0
*HLA-DRA*	HLA-DR α	14.8
*HLA-DRB1*	HLA-DR β1	8.0
**Fcγ receptors**		
*FCGR1*	CD64	1.9
*FCGR2*	CD32	1.4
*FCGR3*	CD16	1.8
**Toll-like receptors**		
*TLR2*	TLR2	2.0
*TLR6*	TLR6	2.9
*TLR8*	TLR8	2.7
*TLR9*	TLR9	3.1
*TLR10*	TLR10	4.3
**Enzymes**		
*PTGS2*	COX-2	5.7
**Cytokines/chemokines/growth factors**		
*CSF2*	GM-CSF	3.5
*CSF3*	G-CSF	9.8
*CXCL11*	CXCL11	5.9
*IL15*	IL-15	6.5
*IL1A*	IL-1α	4.7
*IL1B*	IL-1β	4.6
*IL8*	IL-8	8.4
*TNF*	TNF	2.1

*Target array analysis of unstimulated MΦ(IFNγ)s vs. paired unstimulated MΦ(C)s (*N* = 6)*.

### IFNγ Enhances HSS-Mediated Internalization of *Tp*, Inflammatory Immune Signature, and Cytokine Production by Human Macrophages

**Figure 4:**
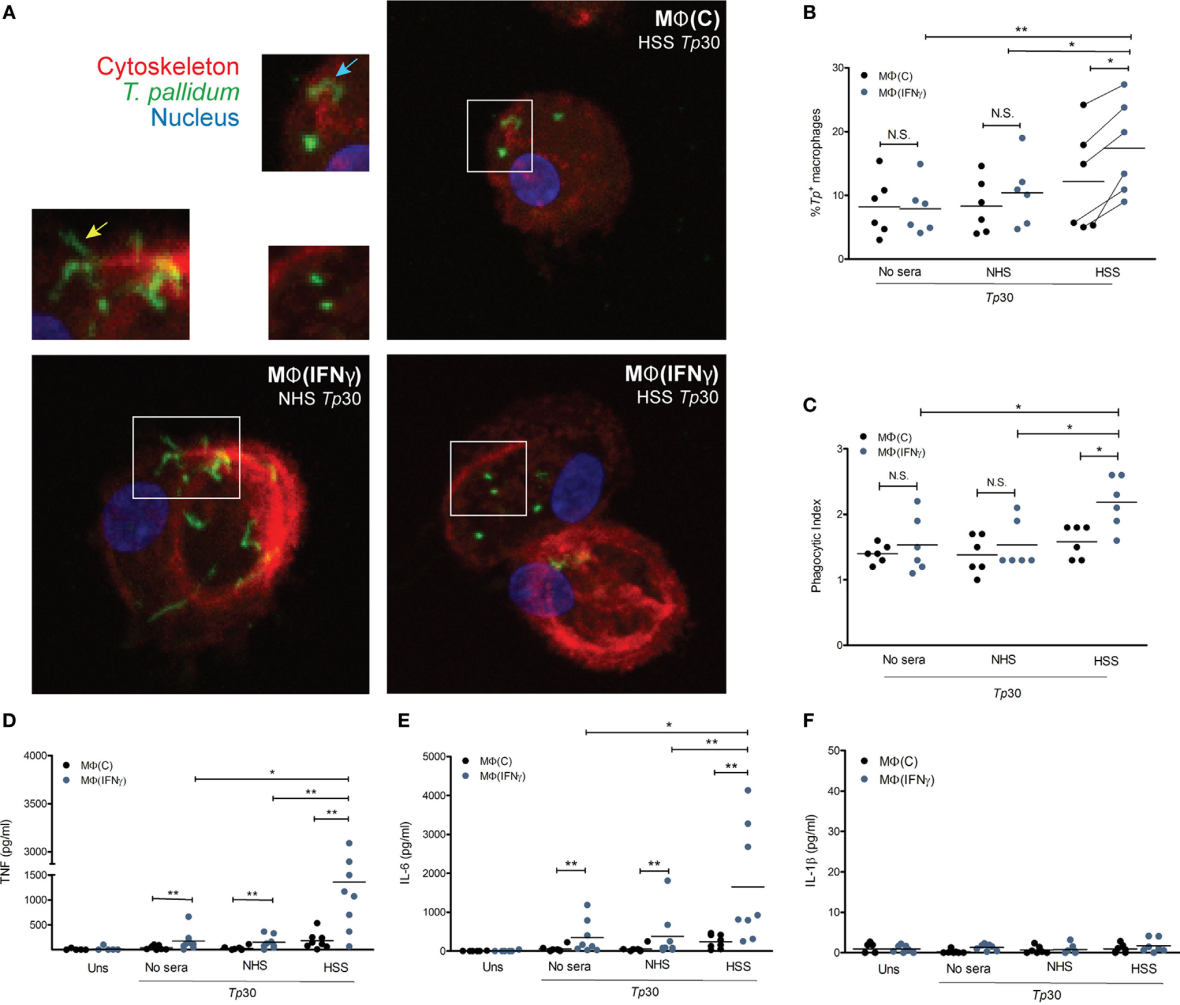
*Treponema pallidum* (*Tp*) uptake by IFNγ activated MΦs. MΦ(C)s and MΦ(IFNγ)s were stimulated with MOI 30:1 of *Tp* for 8 h. *Tp* were either incubated alone (no sera) or where indicated with 10% heat inactivated normal human serum (NHS) or human syphilitic serum (HSS). **(A)** Following stimulation, macrophage cytoskeleton (red), *Tp* (green), and nucleus (blue) were labeled as described in Section “Materials and Methods.” Representative confocal micrograph of NHS-*Tp* (Lower left panel, and left inset) show spirochetes binding by tip attachment as well as laying entirely against the cell surface. Confocal micrographs are a composite display of 20 consecutive Z-stack planes. Internalization of *Tp* was observed with HSS as shown in the two right panels. Quantification of spirochetal uptake was calculated by **(B)** % *Tp*^+^ macrophages and **(C)** phagocytic index. Phagocytosis of *Tp* was significantly enhanced by both HSS and IFNγ. Cytokine bead array was used to detect inflammatory cytokines in the culture supernatants following stimulation with *Tp*. **(D)** TNF and **(E)** IL-6 were significantly increased by MΦ(IFNγ)s following antibody-mediated uptake of *Tp*. **(F)** No IL-1β was detected in any experimental condition. The statistical significance between the *Tp* conditions was determined by Friedman’s test with a Dunnett’s multiple comparison post-test analysis. N.S., not significant, *p-value of <0.05, **p-value of <0.01, ***p-value of <0.001.

Having generated macrophages by closely considering the microenvironment of syphilitic skin lesions, we then asked how these cells would respond to opsonized *Tp ex vivo*. To address this question, we stimulated MΦ(C)s and MΦ(IFNγ)s with *Tp*, in the presence or absence of opsonic antibodies, and assessed their phagocytic potential and immune responsiveness by confocal microscopy and cytokine production, respectively. We observed binding of the spirochete to both macrophage phenotypes, irrespective of the presence of HSS (Figure S4A in Supplementary Material). Spirochetes were seen attached to the macrophages by their tips (Figure 4A, bottom left inset—yellow arrow) and in some cases across the entire length of the bacterial surface (Figure 4A, top right inset—blue arrow). The addition of HSS led to a marked increase in phagocytosis of *Tp* by both macrophage phenotypes (Figures 4A,B), although the phagocytic index was significantly greater in IFNγ stimulated cells (Figure 4C). In agreement with enhanced uptake of opsonized spirochetes, we recovered significantly fewer spirochetes when HSS was included in the stimulation experiments (Figure S4B in Supplementary Material). *Tp* stimulated MΦ(IFNγ)s secreted more TNF (Figure 4D) and IL-6 (Figure 4E) than their counterparts without IFNγ, but neither macrophage phenotype secreted IL-1β (Figure 4F). Overall, these findings confirm our hypothesis that elements present within the syphilitic tissue microenvironment, specifically IFNγ, alter macrophage uptake and responsiveness to opsonized *Tp*.

**Figure 5:**
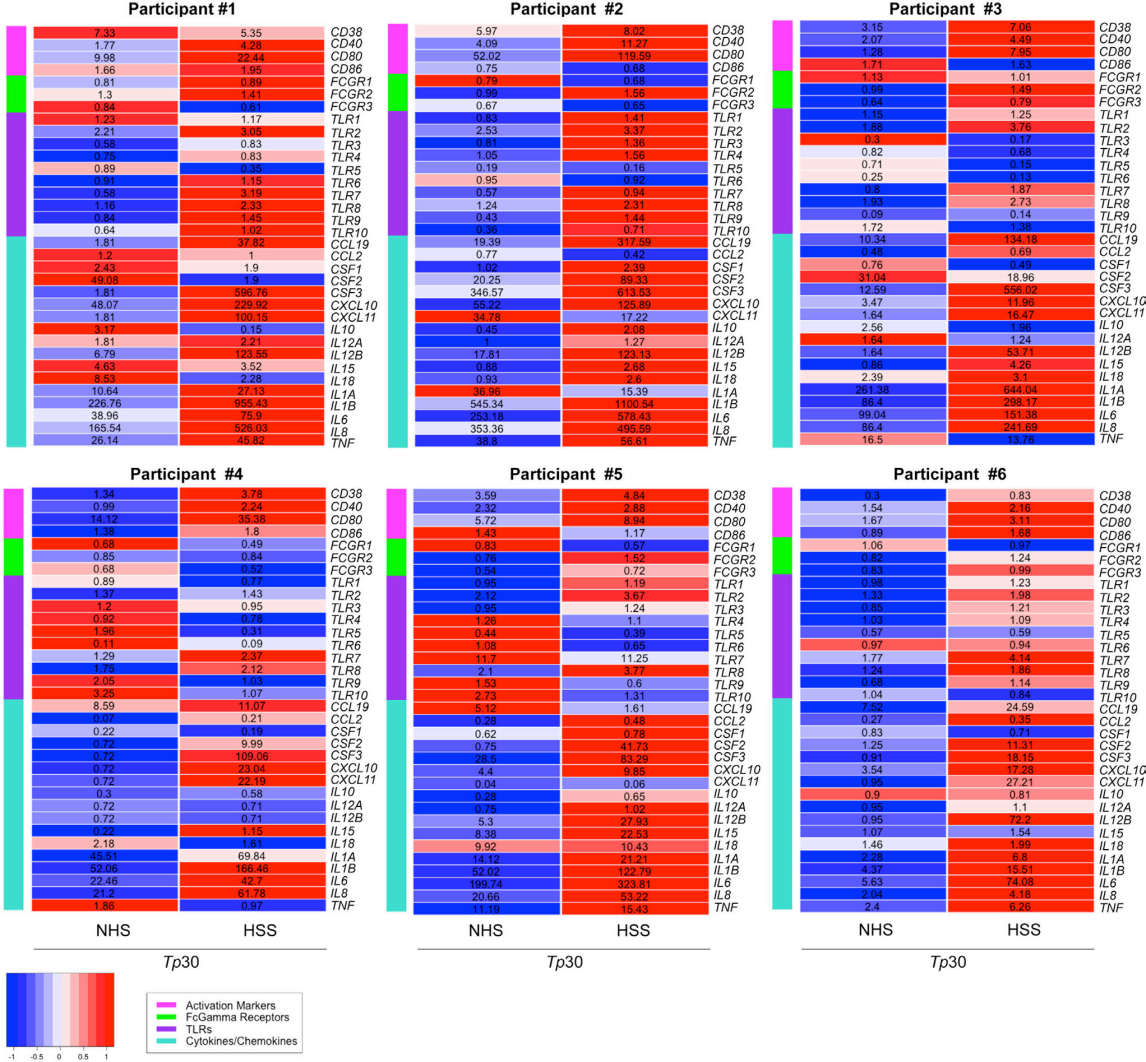
Transcriptional profile of MΦ(IFNγ) stimulated with *Treponema pallidum* (*Tp*). Transcription profiles were determined by targeted array transcriptional analysis after stimulation of MΦ(IFNγ)s with unopsonized [no sera or normal human serum (NHS)] or opsonized [human syphilitic serum (HSS)] *Tp* (MOI 30:1) for 8 h in six-well tissue culture treated plates. Relative fold changes were normalized to the unstimulated MΦ(IFNγ)s control for each gene and then heat maps were generated based on gene categories of interest: activation markers, Fcγ receptors (FcγRs), TLRs, and cytokines/chemokines. Heat maps depict the *Z*-score of NHS-*Tp*30 and HSS-*Tp*30, based on comparing all stimulation conditions, with relative fold-changes values in corresponding gene panel. Each heat map represents an *N* = 1 for individual participants, from a total of six independent experiments.

To further characterize the effect of opsonized *Tp* on the macrophage, we used targeted phagocytic and inflammatory transcriptional array analysis. For these experiments, the relative fold changes of gene transcripts between *Tp* stimulated and unstimulated MΦ(IFNγ)s were measured in three experimental conditions; *Tp* with no sera, with NHS and with HSS (Figure 5). We first compared genes induced in *Tp* stimulated MΦ(IFNγ)s with no sera or with NHS. Consistent with our findings above, where spirochetal uptake and cytokine secretion were similar between the two conditions in the absence of HSS, we observed that transcriptional profiles were also comparable, such that none of the genes met our differential expression threshold of two-fold change and *p*-value of <0.05 (Figure S5A in Supplementary Material). On the other hand, several genes were significantly upregulated in *Tp* stimulated MΦ(IFNγ)s in the presence of HSS when compared to similarly stimulated MΦ(IFNγ)s with NHS (Figure S5B in Supplementary Material). *CD38, CD40* and *CD80* were increased, whereas the activation marker *CD86* varied greatly between the individual participants (Figure 5). *TLR7* was markedly increased in 5/6 healthy volunteer macrophages, but this increase was not statistically significant. *TLR2* and *TLR8*, which have been shown to be important in spirochetal recognition (33, 53, 55) were significantly upregulated as a result of FcγR-mediated uptake of *Tp*. Given the significant upregulation of *TLR8* (Figure 5) and the importance of type I IFNs in response to spirochetes (33, 56), in parallel RT-PCR experiments, we also assessed the expression of *IFNB*, and three interferon regulatory factors (IRFs, *IRF3, IRF5*, and *IRF7*) by RT-PCR. We observed no change in expression of *IRF3* and *IRF5* (Figure S5C in Supplementary Material). *IRF7* was significantly upregulated, but we did not detect a substantial change in *IFNB* expression (Figure S5D in Supplementary Material).

We observed that many cytokine and chemokines involved in cellular recruitment as well as induction of other physiological responses, including enhanced phagocytosis, were differentially regulated in *Tp* stimulated MΦ(IFNγ)s in the presence of HSS. Among them, granulocyte macrophage colony-stimulating factor (GM-CSF), which is encoded by *CSF2* and has been described to trigger the differentiation and exiting of monocytes from the bone marrow was upregulated. There was a strong induction of chemotactic factors, including *CXCL10* and *CXCL11*, which are important for recruitment of monocytes/macrophages to the site of infection. *IL12B*, which is known to stimulate T cells and NK cells to secrete IFNγ, was robustly increased in 5/6 healthy volunteer macrophages. The transcript for *IL15* (Figure 5), a strong NK cell activating cytokine (57), was also elevated. Although IL-1β protein was not secreted by the macrophages following *Tp* stimulation (Figure 4D), *IL1B* was transcriptionally upregulated (Figure 5). The findings together suggest that neither *Tp* nor its PAMPs, escape the phagosome into the cytosol to induce caspase activation and cleavage of pro-IL-1β into the active cytokine in accord with the mechanism detailed by Netea et al. (58).

### CD64 Is Primarily Responsible for Macrophage Driven Opsonophagocytosis of *Tp*

**Figure 6:**
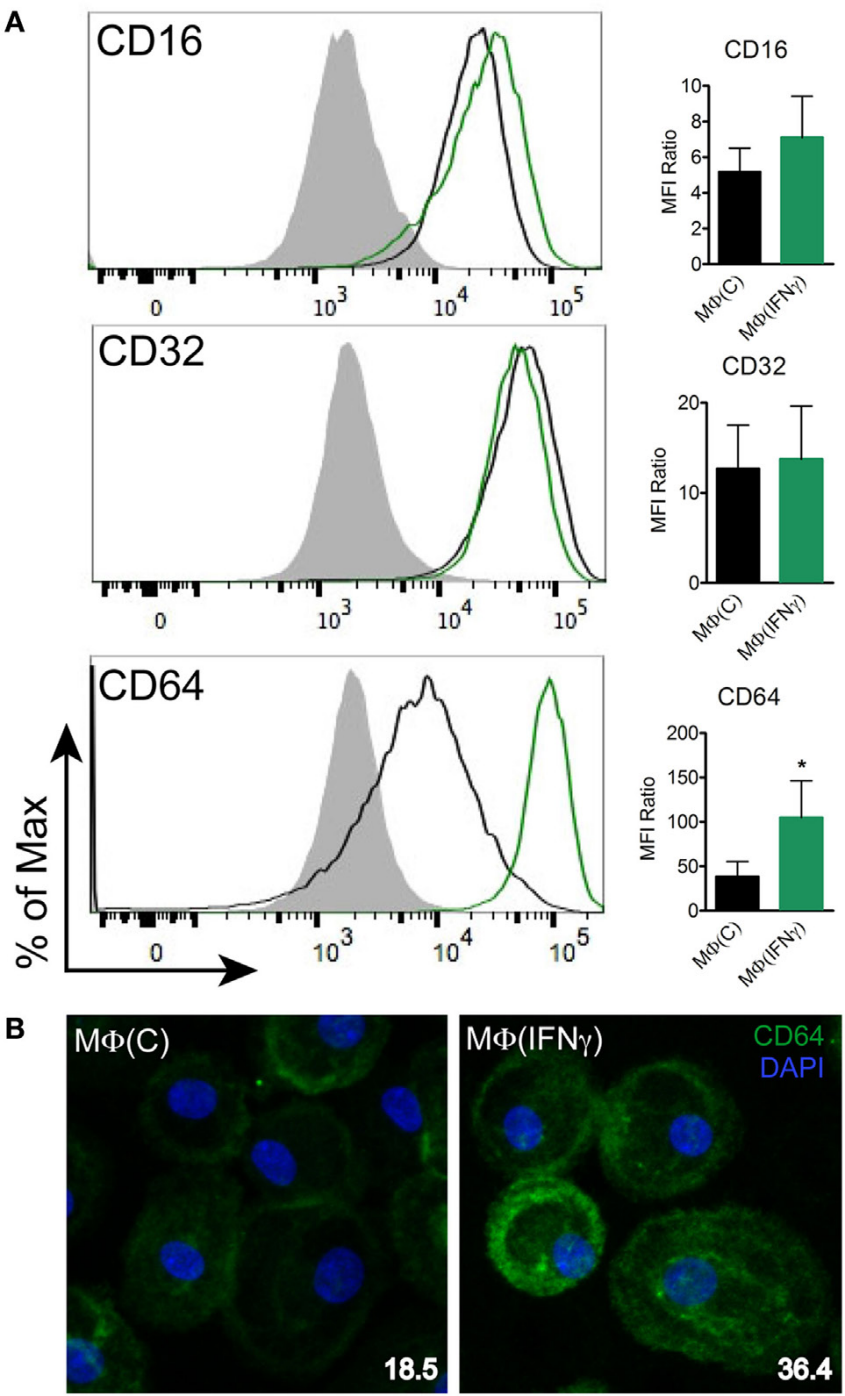
CD64 expression on human macrophages. MΦ(C)s (black) and MΦ(IFNγ)s (green) from the same participant were assessed to determine the expression level of **(A)** Fcγ receptors (FcγRs): CD16, CD32, and CD64 by flow cytometry. The filled gray histograms indicate the isotype control. Histograms are representative of a minimum of four independent experiments. Data of the mean fluorescence intensity (MFI) ratio ± the SD were analyzed for statistical significance using the paired Student’s *t*-test, *p-value of <0.05. **(B)** MΦ(C)s and MΦ(IFNγ)s were incubated in eight-well chamber slides and expression of CD64 (green) was determined by IFA with mouse anti-human CD64 (clone 10.1) antibody. Representative confocal micrographs of CD64 expression levels shown as a composite of 10 consecutive Z-stack planes. CD64 MFI values (inset in bottom right) were calculated using ImageJ.

Fcγ receptors have been observed to be upregulated in syphilitic skin lesions by transcriptional analysis (10) and are important for binding of IgG with various receptor–ligand affinities (59). Based on our primary human macrophage data (Figures 2A and 4A) and published studies (10, 12, 19, 23) demonstrating the importance of HSS in spirochetal uptake, we hypothesized that FcγRs are the major phagocytic receptors for *Tp*. To study this premise, we first assessed the effect of IFNγ on CD16, CD32, and CD64 expression by flow cytometry. Expression of CD16 (FcγRIII), which weakly binds IgG (59), was only mildly affected by IFNγ, while expression of CD32 (FcγRII), another low affinity IgG binder, was not affected (Figure 6A). Conversely, expression of CD64 (FcγRI), a high affinity receptor which binds IgG, specifically IgG_1_ and IgG_3_ (59), was significantly upregulated by IFNγ (Figures 6A,B).

**Figure 7:**
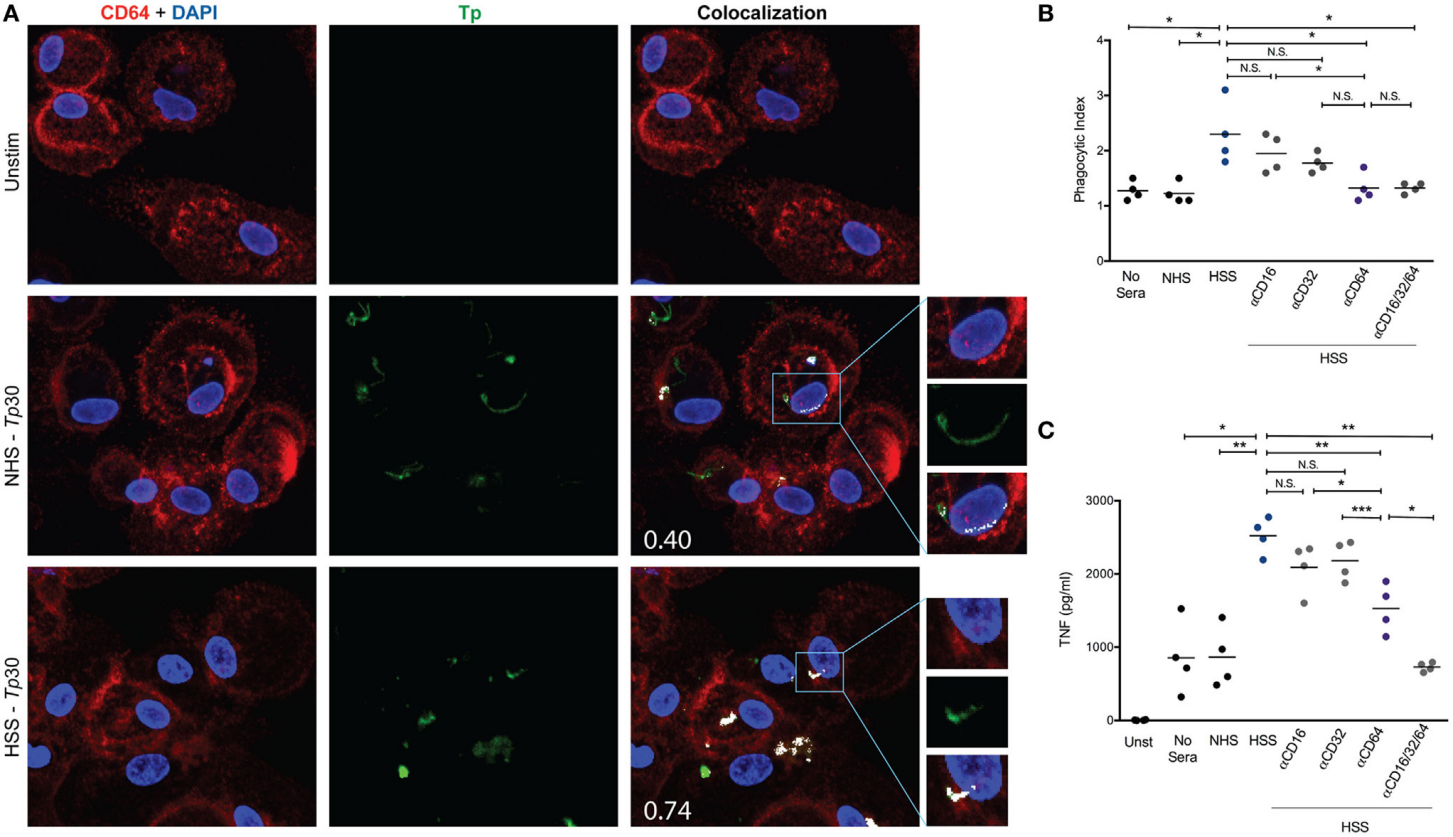
CD64 is the primary phagocytic receptor. Mouse anti-human CD64 and polyclonal rabbit anti-*Tp* antibodies were used to identify locations of Fcγ receptors (FcγRs) and spirochetes on the cell surface by confocal microscopy. **(A)** Colocalization of CD64 (red pixels) and *Tp* (green pixels) were determined by ImageJ plug-in “JACoP” and are represented by white pixels. Mander’s colocalization coefficient values (M2), shown in the lower left corner of colocalization images in A, are indicative of the proportion of the green signal overlapping with the red signal. **(B,C)** The blockade of FcγRs on the surface of MΦ(IFNγ)s was achieved by pre-incubating macrophages with 10 μg/ml of mouse anti-human CD16 (clone 3C8), mouse anti-human CD32 (clone 6C4), mouse anti-human CD64 (clone 10.1), or 10 μg/ml of each of the three antibodies for 1 h prior to *Tp*–macrophage stimulating at an multiplicity of infection (MOI) 30:1 with no sera, normal human serum (NHS), or human syphilitic serum (HSS) for an additional 8 h in an eight-well chamber slide. **(B)** Phagocytic index is shown and compared between each of the five conditions studied. **(C)** Cytokine bead array was used to detect inflammatory cytokine production following stimulation with *Tp* and shown in the dot plot for each of the five conditions studied. The statistical significance between the *Tp* conditions was determined by Friedman’s test with a Dunnett’s multiple comparison post-test analysis. N.S., not significant, *p-value of <0.05, **p-value of <0.01.

Due to the significant increase in CD64 expression induced by IFNγ (Figure 6A) and importance of syphilitic serum in spirochetal uptake (Figure 4C), we then assessed the localization of CD64 with both unopsonized and opsonized *Tp* by confocal microscopy. CD64 could be seen localizing with unopsonized *Tp* (Figure 7A, middle right) however when the spirochetes were opsonized with HSS, the intensity of the colocalization was more robust and is most likely a result of FcγRs clustering to the site of treponeme attachment (Figure 7A, lower right). To determine the role of FcγRs in spirochetal uptake, we used monoclonal antibodies against human CD16, CD32, and CD64 to block the interaction between opsonized spirochetes and each of the three Fc receptors and then compared bacterial uptake and inflammatory cytokine production between blocked and unblocked MΦ(IFNγ)s. As shown in Figure 7B, there were no significant reductions in uptake by blocking CD16 or CD32. However, blockade of CD64 did cause a significant decrease in phagocytosis of opsonized spirochetes (Figure 7B), as well as TNF production (Figure 7C). This finding was not surprising because IgG1 and IgG3, the prominent IgG subclasses in syphilitic serum (60), are high affinity IgG subclasses known to bind CD64. Importantly, the use of monoclonal antibodies against CD64 alone, or in combination with CD16 and CD32, did not result in complete abrogation of phagocytosis (Figure 7B), suggesting that additional phagocytic receptors could have a role in *Tp* uptake. Taken together, these data show for the first time that CD64 acts as the primary phagocytic receptor for FcγR-mediated uptake of opsonized *Tp*, and that IFNγ-mediated enhanced expression of CD64 promotes Fc-receptor–ligand interactions.

## Discussion

Much of what is currently known about the role of the macrophage in syphilis pathogenesis emanates from several decades of *in vivo* and *ex vivo* studies using the rabbit model of infection (15, 16, 19, 20, 22, 61–63). Lukehart and Miller (19) demonstrated that chemically activated rabbit peritoneal macrophages phagocytose *Tp ex vivo* in the presence of syphilis immune rabbit sera. This landmark study was the first to underscore the importance of the macrophage in Ab-mediated uptake of the syphilis spirochete. Additional rabbit studies established that clearance of the spirochete *in vivo* temporally correlates with the influx of macrophages to infected tissues (15, 16, 64) and generation of a “lymphocyte factor” (65), now known to be IFNγ (18). Although the rabbit model has proved to be an important resource in syphilis research, humans are the obligate host for *Tp*. Thus, it is critical to develop an *ex vivo* human model to aid in understanding the immunological responses of the natural host evoked by the bacterium. Studies to understand the role of the macrophage in human syphilis have relied on a combination of transcriptional and IHC analysis of early syphilitic lesions and are only now being explored with an *ex vivo* macrophage system. Only a handful of studies have evaluated human monocyte/macrophage-*Tp* interactions *ex vivo* (10, 12, 23). In one such study, our group showed that HSS promotes spirochetal uptake by isolated human monocytes, leading to destruction of the bacterium within phagosomal vacuoles and enhanced secretion of proinflammatory cytokines (i.e., TNF) (10). Recently Marra et al. described that 20–47% of monocyte-derived-human macrophages internalized HSS opsonized spirochetes (23). However, the researchers did not assess the impact of the macrophage phenotype in phagocytosis or the inflammatory response elicited to the bacterium. The immunological environment provides critical information when developing a model system because macrophage phenotypic plasticity and polarization *ex vivo* are highly dependent on the cytokines and growth factors used in the differentiation protocols (28, 44, 52, 66). We elected to carefully model the immunologic niche where macrophage–*Tp* interactions are likely to occur under actual disease conditions for our system. We observed that the addition of IFNγ enhanced the macrophage’s phagocytic capacity for opsonized spirochetes, as well as the secretion of inflammatory cytokines. Unlike the rabbit model, where 63–76% of the macrophages contained ingested opsonized spirochetes (19), the human system resulted in a much lower % of *Tp*^+^ cells and confirms the variability of uptake described by Marra et al. (23). We feel this result observed in the *ex vivo* macrophage system more accurately reflects the true duality of the disease; this duality can be observed in Figure 1, where treponemes appear to be migrating to locations that are difficult for the immune cells such as CD68^+^ macrophages to access, while increasing the odds for bacterial transmission.

Our study results reinforce our underlying hypothesis that macrophage differentiation is an important factor for optimal treponemal recognition. In the presence of M-CSF and IFNγ macrophages exhibited a “classically activated” phenotype (67) and responded to opsonized *Tp* by secreting large quantities of TNF. By comparison, while non-IFNγ stimulated macrophages were also capable of internalizing opsonized spirochetes, their cytokine responsiveness was markedly decreased. The difference in cytokine responses between the two macrophage phenotypes has several potential explanations. First, IFNγ induced expression of CD64 is likely to engender more efficient uptake of opsonized spirochetes, which translates into an increase in bacterial cargo available for signaling from within the phagosome. Second, upregulation of baseline TLR2 and TLR8 expression by IFNγ, as shown herein, could promote more opportunities for ligation of released spirochetal PAMPs (33, 53) and TLR signaling itself may lead to a more rapid maturation of the phagosome (68). Alternatively, as shown by Balce et al. (69) FcγR-mediated phagocytosis in association with IFNγ could facilitate phagosomal processing of proteins. IFNγ stabilizes MyD88 (70), in addition to modulation of the FcγR signaling cascade (59, 71), helping to enhance the recognition response to *Tp*. Intriguingly, a similar decrease in cytokine production was not seen in non-IFNγ primed *Bb*-stimulated macrophages. Several reasons likely account for the differential response between *Bb* and *Tp*. Although both spirochetes contain lipoproteins known to signal through TLR2 (33, 55, 72), *Bb* is larger in size than *Tp* and has a far richer repertoire of lipoproteins within its outer and inner membranes. In addition, *Bb*’s nucleic acid is far more abundant and complex than *Tp*’s, thus nucleic acid ligands are likely to have more opportunities to engage their cognate endosomal TLR receptors (i.e., TLR8). Ultimately, macrophages phagocytose *Bb* directly *via* the phagocytic receptor CR3 (42, 73). The process does not require the presence of complement or opsonic antibodies, leads to efficient uptake of the bacterium and activation of phagosomal receptors despite the absence of IFNγ.

Macrophage recognition of a bacterial pathogen is a very dynamic and complex process that involves a variety of receptors and is greatly influenced by the molecular composition and structure of the organism they encounter. Although *Tp* contains an abundance of highly antigenic hydrophilic polypeptides, these molecules are tethered by covalently bound N-terminal lipids to the bacterium’s periplasmic inner membrane leaflet and thus not available for innate immune receptor (i.e., TLR1/2) sensing (74–76). In addition, because *Tp* contains very few OMPs (77, 78), the bacterium provides limited binding sites for syphilitic opsonic antibodies (79). Nevertheless, our study demonstrates that HSS is able to opsonize *Tp* and lead to FcγR-mediated uptake by human macrophages, where CD64 acts as the primary phagocytic receptor. The *Tp*-specific antibodies generated by the host, are primarily comprised of IgG1 and IgG3 subclasses (60), are known to bind to CD64 with high affinity (59). Clustering of CD64, as demonstrated occurs in the presence of opsonized spirochetes (Figure 7A), initiates important signal transduction events that result in internalization of the pathogen (80). Although receptor dimerization can be sufficient for receptor activation, beads coated with low densities of IgG, similar to what occurs with *Tp* (77, 79), trigger inefficient receptor recruitment, thus slowing formation of the phagocytic cup (81). The heterogeneity of antibodies binding to spirochetal populations (79), in combination with the low density of OMPs (77) and fixed locations of the antigenic targets (78) may contribute directly to the slow phagocytic events of the treponemes previously described (82, 83). They may also explain why a large proportion of spirochetes are capable of avoiding opsonophagocytosis (Figure S3B in Supplementary Material). IFNγ-enhanced expression of CD64 most likely engenders more efficient uptake of opsonized spirochetes and inflammatory responses, which allow the human host to override the gridlock caused by the organisms unique OM structure and requirement of opsonic antibodies to eventually clear the pathogen.

Following opsonophagocytosis of a delicate pathogen, such as *Tp*, the acidic environment of the phagosomal vacuole disrupts the spirochete’s OM (12). Liberation of once concealed spirochetal PAMPs makes them accessible to interact with their cognate receptors and initiate downstream inflammatory signals. Herein, we demonstrate that in addition to NF-κB mediated cytokine production, opsonized *Tp* induces transcription of type I interferons, several chemokines, and many other inflammatory mediators. Among them is IFN-β, a type I interferon known to be present in SS skin lesions (10), which we believe is triggered similarly to *Bb, via* a TLR8-IRF7 pathway (53). The chemokines CCL19, CXCL10, and CXCL11, are chemokines associated with recruitment of DCs, antigen engaged B cells, NK cells, and activated T cells to infected tissues (84–86) were differentially transcribed following uptake of opsonized *Tp*, and were upregulated in SS skin lesions (10). IL-12 and IL-15, two cytokines secreted by macrophages and associated with activation of T cells and NK cells (57), were also transcriptionally modulated. Interestingly, activated NK cells and CD8^+^ T cells, known for production of IFNγ in response to *Tp* (12, 14), are typically associated with degranulation in response to viral infection and tumor cells (87, 88) but *Tp* is an extracellular pathogen. The immunological functions elicited by CD8^+^ T cells and NK cells in the syphilitic lesions are unclear, with the exception of sourcing IFNγ for macrophage activation. It is also possible that macrophages are presenting antigen to T cells *via* a cross presentation mechanism and that other lymphocytes are involved in macrophage-independent mechanisms of clearance in the tissues. In short, it is clear that in the context of phagosomal signaling, the macrophage plays a fundamental role in generating proinflammatory signals in response to *Tp*, and also modulates innate and adaptive immune responses.

**Figure 8:**
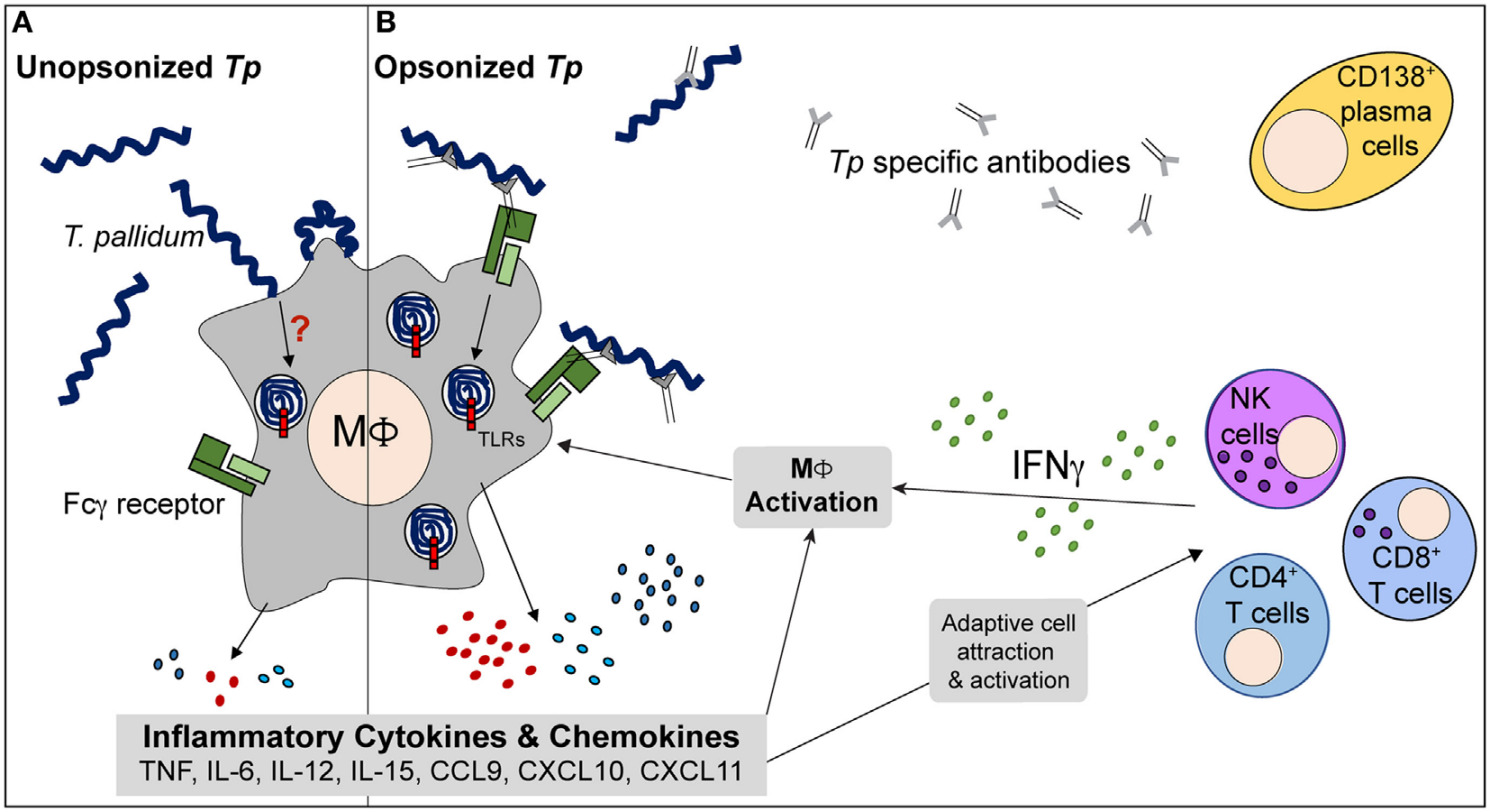
Proposed model of immune interplay in secondary syphilis (SS) lesions. **(A)** During early response to *Tp*, human macrophages (MΦs) are able to bind unopsonized spirochetes but the cells phagocytose very limited numbers and many *Tp* are able to escape. The phagocytic process is inefficient and results in minimal inflammatory cytokine production. **(B)** Following antigen presentation by phagocytes, lymphocytes such as CD4^+^ T cells, CD8^+^ T cells, and NK cells produce IFNγ locally. T cell activation can aid in B cell maturation and *Tp* specific Ab production. The antibodies are directed to various targets of the treponemes, including the spirochete’s rare outer membrane proteins. MΦs increase expression of Fcγ receptors (FcγRs) in response to the IFNγ rich microenvironment of the infected tissue and allow for enhanced phagocytosis of opsonized treponemes. Within the MΦs’ phagosomal compartment, the spirochetes’ fragile outer membrane is degraded, liberating the once concealed pattern-associated molecular patterns (PAMPs) and ultimately resulting in interaction with TLRs, such as TLR2 and TLR8. The activated MΦs produce elevated levels of inflammatory cytokines (TNF, IL-12, and IL-15) and chemokines, resulting in an important positive feedback loop that acts on both branches of the immune system. Ultimately, with aid from the adaptive immune system, the macrophage plays a central role in clearance of *Tp*.

Venereal syphilis can be considered a battle between the ability of *Tp* to circumvent immune recognition and the proficiency of the host’s innate and adaptive immune responses to search and destroy the spirochetal pathogen (38, 39, 89). Our results reinforce the importance of the human macrophage as a contributor in the innate immune response to *Tp*. They also offer new evidence that the balance between phagocytic uptake of the spirochete and the bacterium’s ability to evade immune recognition by the macrophage is significantly influenced by the emergence of anti-treponemal opsonic antibodies, as well as the immune microenvironment where the macrophage resides. More specifically, our findings provide unequivocal confirmation that HSS markedly enhances uptake of spirochetes by human macrophages *in vitro*. Of particular noteworthiness, our study is the first to show that in human macrophages CD64 is the primary receptor during FcγR-mediated phagocytosis of *Tp*. Results from this study also substantiate the importance of IFNγ-mediated macrophage activation as a beneficial immune event that tips the balance of the battle with the spirochete in favor of the host. These findings allow us to build upon the model initially proposed by Lukehart (90) for the role of the human macrophage in the immunologic events that take place in the context of early syphilitic infection in humans. As shown in the model (Figure 8), phagocytosis by macrophages is central to the immune response against *Tp* and greatly influenced by Ab production to specific antigenic epitopes and production of IFNγ. The model proposes that following a prolonged period of time, and many struggles to gain control of the spirochete, macrophage-dependent and -independent immune responses ultimately lead to clearance of *Tp* from infected tissues. Our study also indicates the potential challenges faced in the development of a vaccine against a unique bacterium that avoids host recognition. Comprehensive analysis of confirmed OMPs such as TprC, TprD, and Tp0326 by our group (75, 76, 91) in addition to other proteins identified by other researchers (92–94), will be critical to better understand the surface-exposed antigenic loops of *Tp* that antibodies utilize for opsonization. Lastly, our *ex vivo* human macrophage model will provide researchers with the first human system to assess surrogate markers for syphilis vaccine candidates.

## Ethics Statement

This study was carried out in accordance with the recommendations of the Institutional Review Boards at UConn Health, Farmington CT and Centro Internacional de Entrenamiento e Investigaciones Médicas (CIDEIM) in Cali, Colombia. All study participants were provided written informed consent. All animal experimentation was conducted following the Guide for the Care and Use of Laboratory Animals (8th Edition) and in accordance with protocols reviewed and approved by the UConn Health Institutional Animal Care and Use Committee under the auspices of Animal Welfare Assurance A347-01.

## Author Contributions

All authors contributed to the conception, design, and analysis of experiments. KH, AC, CLV, ML, LR, and DM performed the experiments in the manuscript. KH, JR, and JS wrote the manuscript. All authors critically reviewed the manuscript for intellectual content.

## Conflict of Interest Statement

The authors declare that the research was conducted in the absence of any commercial or financial relationships that could be construed as a potential conflict of interest.
