# Supplementary material for: IFNγ Enhances CD64-Potentiated Phagocytosis of Treponema pallidum Opsonized with Human Syphilitic Serum by Human Macrophages
Source: Front Immunol. 2017 Oct 5;8:1227. doi: 10.3389/fimmu.2017.01227 (PMC5633599; doi:10.3389/fimmu.2017.01227)
Supplement: Supplementary file 1 [file Presentation_1.PDF]

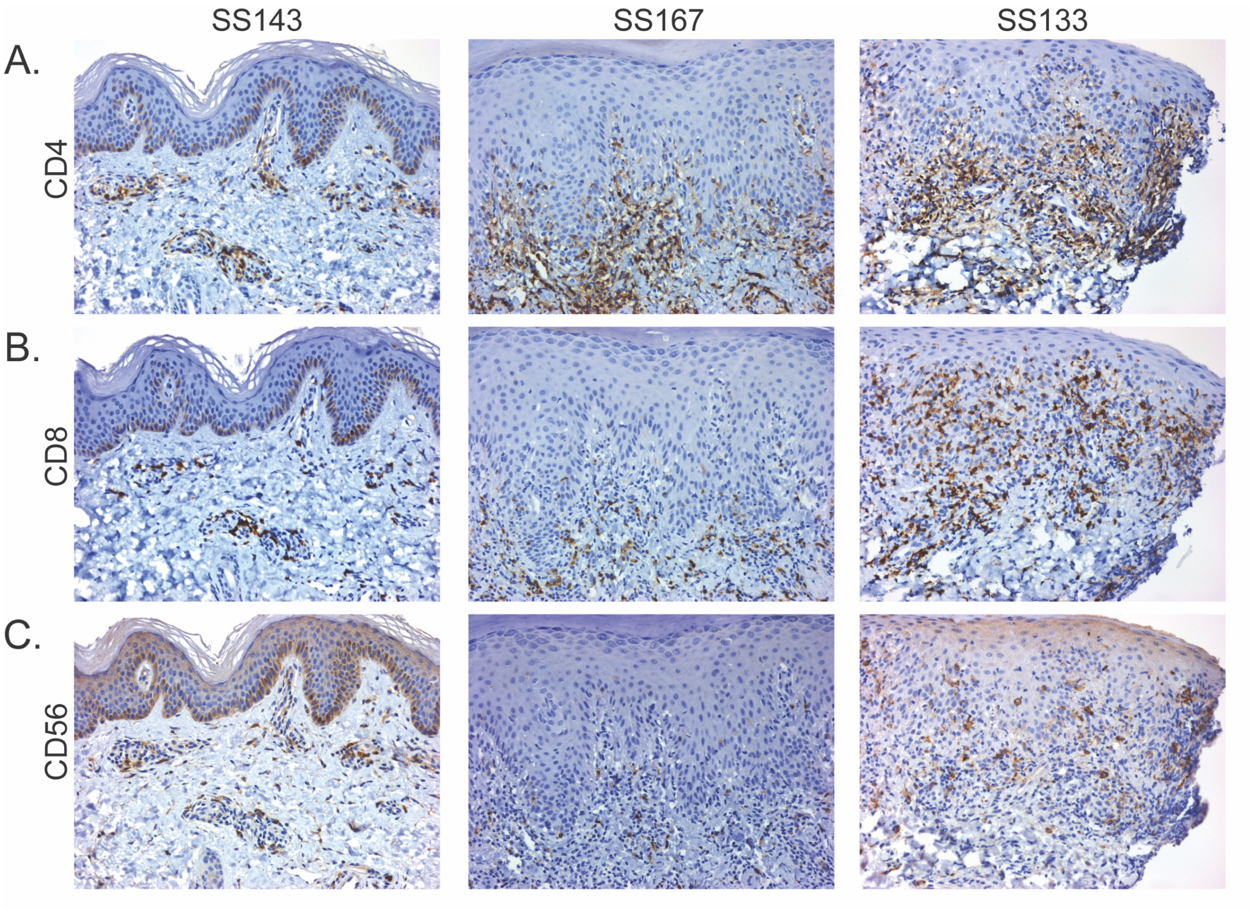

Supplemental figure 1. Lymphocytic infiltration of secondary syphilis skin lesions.

Representative skin biopsies obtained from three secondary syphilis (SS) patients' skin lesions were processed for IHC analysis of **(A)** $CD4^+$ **(B)** $CD8^+$ and **(C)** $CD56^+$ cells (20x magnification).

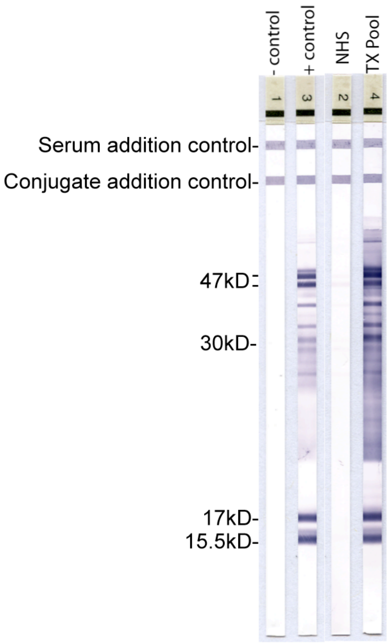

Supplemental figure 2. Pooled syphilitic serum is reactive with *T. pallidum* proteins

Reactivity of pooled sera from five HIV-negative syphilis patients (HSS) against *Tp* proteins was determined by Trinity IgG immunoblot.

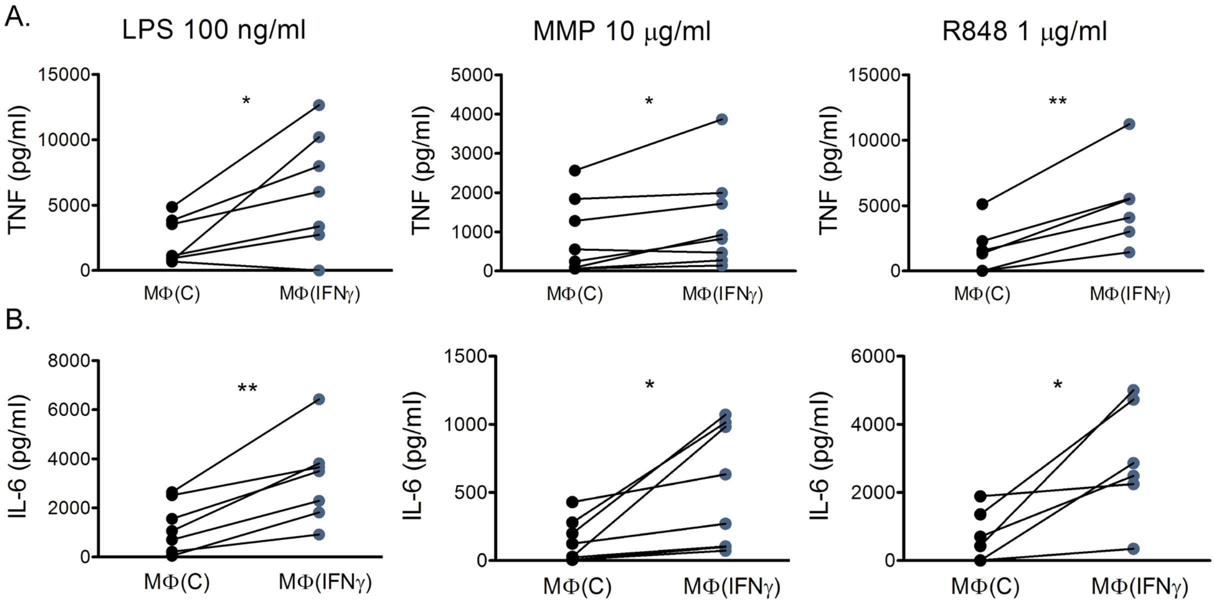

Supplemental figure 3. NFκB-mediated cytokine response to TLR ligands

Paired MΦ(C)s (black circle) and MΦ(IFNγ)s (blue circle) were stimulated with ligands for TLR4 (100 ng/ml of LPS), TLR2 (10 μg/ml of MMP) and TLR7/8 (1 μg/ml of R848) for 8 h. Following stimulation, supernatants were analyzed by cytokine bead array. The MΦ(IFNγ)s secreted significantly more **(A)** TNF and **(B)** IL-6 as a result of TLR stimulation. N=6 to 8 independent experiments, statistical analysis by paired Student's *t*-test *p-value of < 0.05, ** p-value of < 0.01.

A.

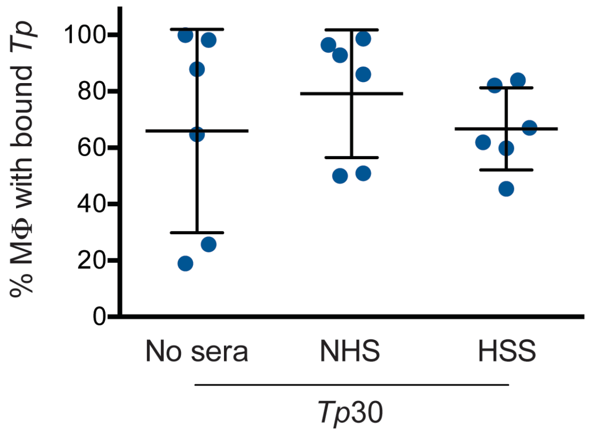

B.

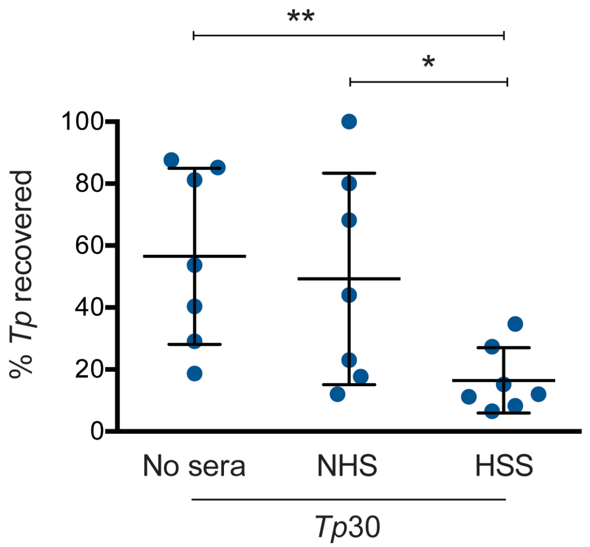

Supplemental figure 4. *T. pallidum* bind to MΦ(IFNγ)s but a large percentage is still recovered, even in the presence of HSS.

MΦ(IFNγ)s were stimulated with MOI 30:1 of *Tp* for 8 h. *Tp* were either incubated alone (No sera) or where indicated with 10% heat inactivated NHS or HSS. Following stimulation, macrophage cytoskeleton, *Tp* and nucleus were detected as described in “Material and methods”. **(A)** The percentage of MΦ(IFNγ)s with bound HSS *Tp* was determined to be reduced by assessing interaction of spirochetes with the cell surface in six independent experiments. **(B)** The percentage of HSS opsonized spirochetes recovered from the supernatant of MΦ(IFNγ)s stimulations were

significantly reduced. Statistical significance was assessed by a paired Student's *t*-test, *p-value of < 0.05, **p-value of < 0.01.

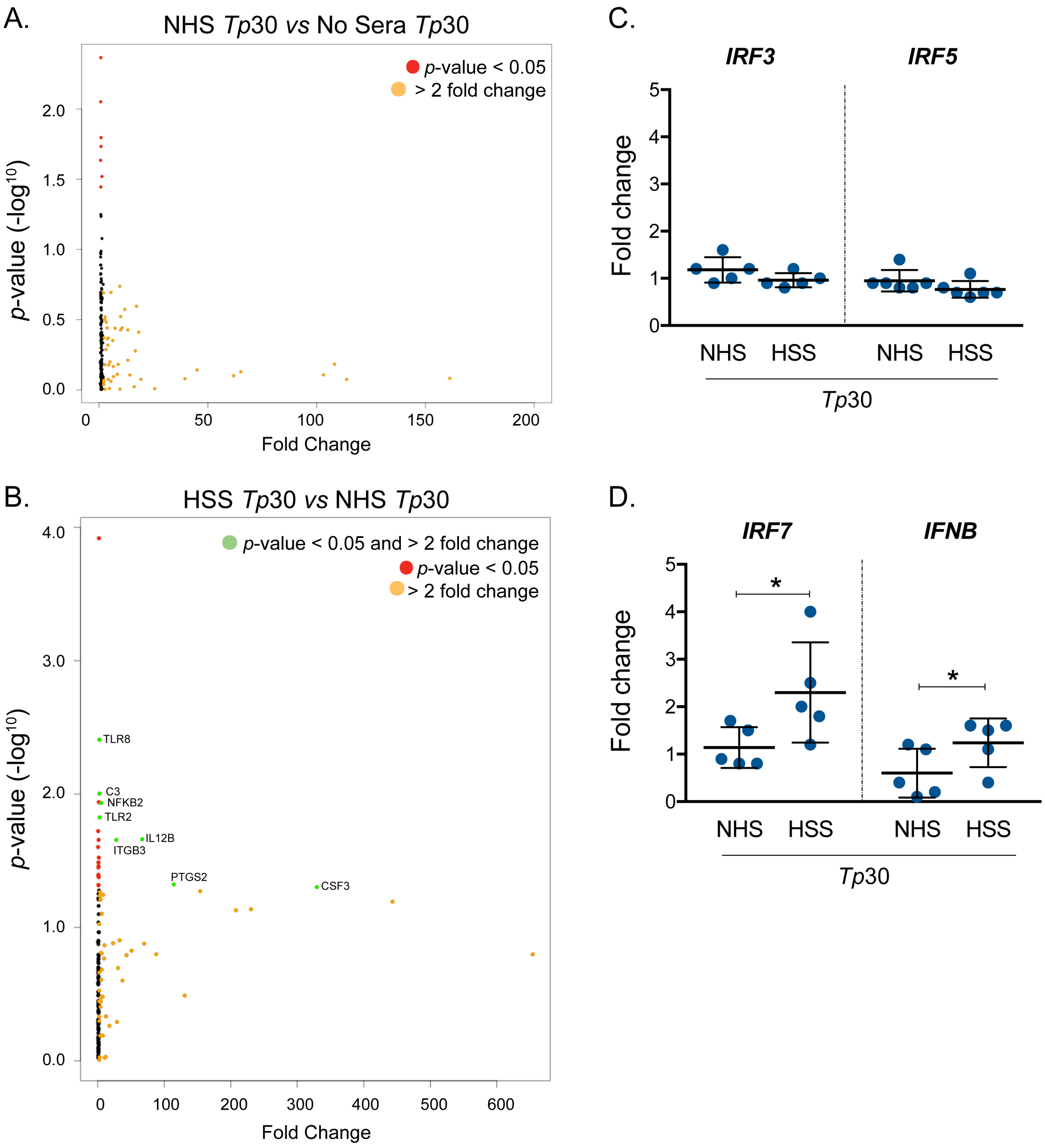

Supplemental figure 5. Transcriptional profiles of MΦ(IFNγ)s following stimulation with *T. pallidum.*

Transcription profiles were determined by targeted array transcriptional analysis after stimulation of MΦ(IFNγ)s with unopsonized (No sera or NHS) or opsonized (HSS) *Tp* (MOI 30:1) for 8 h in six-well tissue culture treated plates. Relative fold changes were normalized based on the

unstimulated MΦ(IFNγ) control for each gene, N=6 independent experiments. Statistical significant was determined by a paired Student's *t*-test. The relative fold change and *p*-value of each gene was plotted by volcano plot comparing **(A)** No Sera *Tp* vs NHS *Tp* and **(B)** NHS *Tp* vs HSS *Tp*. Transcription of **(C)** IRF3, IRF5, **(D)** IRF7 and IFN-*β* was determined by RT-PCR following *Tp* stimulation MΦ(IFNγ)s. Data of the MFI ratio ± the S.D. were analyzed for statistical significance using the paired Student's *t*-test, *p-value of < 0.05.

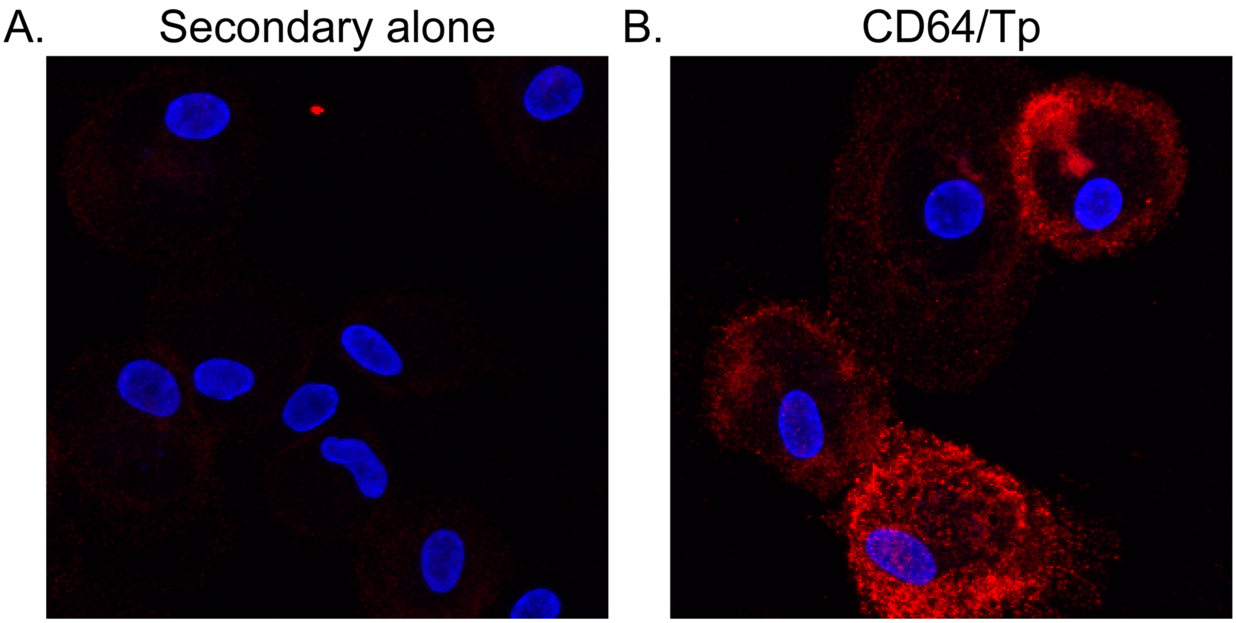

Supplemental figure 6. CD64 and *Tp* IFA controls

Representative confocal micrographs of CD64 and *Tp* expression levels shown as a composite of 12 consecutive Z-stack planes. Mouse anti-human CD64 and polyclonal rabbit anti-*Tp* antibodies were used to identify locations of FcγRs and spirochetes on the cell surface by confocal microscopy. (**A**) Secondary antibody alone controls and (**B**) unstimulated macrophages demonstrate antibody specificity. Red: CD64, green: *T. pallidum*, blue: nucleus.
